# An adaptive signaling network in melanoma inflammatory niches confers tolerance to MAPK signaling inhibition

**DOI:** 10.1084/jem.20160855

**Published:** 2017-06-05

**Authors:** Helen L. Young, Emily J. Rowling, Mattia Bugatti, Emanuele Giurisato, Nadia Luheshi, Imanol Arozarena, Juan-Carlos Acosta, Jivko Kamarashev, Dennie T. Frederick, Zachary A. Cooper, Alexandre Reuben, Jesus Gil, Keith T. Flaherty, Jennifer A. Wargo, William Vermi, Michael P. Smith, Claudia Wellbrock, Adam Hurlstone

**Affiliations:** 1 Manchester Cancer Research Centre, Faculty of Biology, Medicine, and Health, School of Medical Sciences, Division of Molecular and Clinical Cancer Studies, The University of Manchester, Manchester M13 9PT, England, UK; 2 Department of Molecular and Translational Medicine, Section of Pathology, University of Brescia, 25123 Brescia, Italy; 3 Department of Molecular and Developmental Medicine, University of Siena, 53100 Siena, Italy; 4 Division of Oncology, MedImmune Ltd, Cambridge CB21 6GH, England, UK; 5 Edinburgh Cancer Research Centre, Medical Research Council Institute of Genetics and Molecular Medicine, Western General Hospital, Edinburgh EH4 2XR, Scotland, UK; 6 Department of Dermatology, University Hospital Zürich, 8091 Zürich, Switzerland; 7 Department of Medicine, Massachusetts General Hospital Cancer Center, Boston, MA 02114; 8 Division of Surgical Oncology, University of Texas MD Anderson Cancer Center, Houston, TX 77030; 9 Medical Research Council London Institute of Medical Sciences, London W12 0NN, England, UK; 10 Institute of Clinical Sciences, Faculty of Medicine, Imperial College London, London W12 0NN, England, UK; 11 Department of Pathology and Immunology, Washington University School of Medicine, St. Louis, MO 63110

## Abstract

Mitogen-activated protein kinase (MAPK) pathway antagonists induce profound clinical responses in advanced cutaneous melanoma, but complete remissions are frustrated by the development of acquired resistance. Before resistance emerges, adaptive responses establish a mutation-independent drug tolerance. Antagonizing these adaptive responses could improve drug effects, thereby thwarting the emergence of acquired resistance. In this study, we reveal that inflammatory niches consisting of tumor-associated macrophages and fibroblasts contribute to treatment tolerance through a cytokine-signaling network that involves macrophage-derived IL-1β and fibroblast-derived CXCR2 ligands. Fibroblasts require IL-1β to produce CXCR2 ligands, and loss of host IL-1R signaling in vivo reduces melanoma growth. In tumors from patients on treatment, signaling from inflammatory niches is amplified in the presence of MAPK inhibitors. Signaling from inflammatory niches counteracts combined BRAF/MEK (MAPK/extracellular signal–regulated kinase kinase) inhibitor treatment, and consequently, inhibiting IL-1R or CXCR2 signaling in vivo enhanced the efficacy of MAPK inhibitors. We conclude that melanoma inflammatory niches adapt to and confer drug tolerance toward BRAF and MEK inhibitors early during treatment.

## Abstract (precis)

Drug tolerance brought about by reversible adaptive responses precedes the emergence of irreversible mutation-driven drug resistance and sustains tumor cells when at their most vulnerable. Young et al. delineate a signaling relay incorporating IL-1 and CXCR2 ligands emanating from melanoma-associated macrophages and fibroblasts, respectively, that confer tolerance to MAPK inhibitors.

## Introduction

Melanoma cells rely heavily on extracellular signal–regulated kinase (ERK)/MAPK signaling as indicated by hyperactivation of this pathway in up to 90% of melanomas. The MAPKKK BRAF is a prominent oncogene in melanoma (Davies et al., 2002), and inhibitors that target BRAF^V600E^, the most commonly mutated form, are extremely potent, eliciting high response rates (Flaherty et al., 2010; Chapman et al., 2011; Sosman et al., 2012). Despite this, durable responses are rare, and most patients relapse within a year after commencement of treatment (Salama and Flaherty, 2013). Significantly longer responses can be achieved by combining BRAF inhibitors (BRAFi’s) and MEK (MAPK/ERK kinase) inhibitors (MEKi’s), yet the development of drug resistance is still the most common outcome (Long et al., 2016). Acquisition of mutations affecting a variety of components of the RTK-RAS-RAF-MEK-ERK pathway, but also parallel pathways including the PI3K-AKT pathway, enable melanoma cells to resist MAPK signaling inhibition. Moreover, subclones of transformed cells from tumors at distinct anatomical sites, but also within a given tumor, possess different resistance-conferring mutations (Shi et al., 2014; Van Allen et al., 2014; Kemper et al., 2015), and this inter- and intratumoral heterogeneity poses a formidable obstacle to the development of any salvage therapy. Consequently, focus has recently shifted to defining alterations in intracellular signaling, metabolism, chromatin structure, and gene expression that comprise early (hours to weeks) adaptive responses of cells to MAPK pathway inhibitors, which are reversible (that is independent of acquired mutations) and contribute to the ability of transformed cells to tolerate these therapeutic agents before acquired resistance takes hold (Smith and Wellbrock, 2016). Such adaptive responses can occur in a cancer cell–autonomous fashion (Johannessen et al., 2010; Nazarian et al., 2010; Villanueva et al., 2010; Poulikakos et al., 2011; Smith et al., 2013; Long et al., 2014). However, it also appears that factors elaborated by stromal and innate immune cells in the tumor microenvironment also enable melanoma cells to tolerate MAPK inhibition (Straussman et al., 2012; Smith et al., 2014; Hirata et al., 2015; Wang et al., 2015). Potentially, compared with mutation-driven events, tumors’ adaptive responses to drugs may be more stereotypical; simultaneously targeting adaptive responses and MAPK signaling might greatly diminish the burden of residual transformed cells, which could otherwise go on to evolve mutations conferring drug resistance (Smith and Wellbrock, 2016).

Importantly, in melanoma patients undergoing MAPK inhibitor treatment, we have shown previously that there is a greater macrophage abundance within the tumors compared with pretreatment (Smith et al., 2014). Macrophages are the major producers of the proinflammatory cytokine TNF, and we and others have shown that TNF not only is important for melanoma growth and invasion, but also contributes to tolerance to MAPK inhibition (Katerinaki et al., 2003; Gray-Schopfer et al., 2007; Smith et al., 2014). However, TNF is not the only proinflammatory cytokine produced by macrophages, and the increased number of macrophages during treatment with MAPK inhibitors might impact drug efficacy through additional factors. One such factor that is closely linked to TNF and produced by macrophages in abundance is IL-1. IL-1 exists as two isoforms, α and β, which both signal via the IL-1 receptor (IL-1R) and the transcription factor NF-κB. However, whereas IL-1α is widely and constitutively expressed and initiates inflammation when passively released from necrotic cells, IL-1β expression is more restricted. Furthermore, unlike IL-1α, the pro-form of IL-1β requires cleavage by caspase 1, which is, in turn, activated by the NLRP3-containing inflammasome, to become active (Garlanda et al., 2013).

Studies on IL-1 expression in established human melanoma cell lines are inconsistent, ranging from constitutive IL-1β expression and secretion only in metastases-derived cells (Okamoto et al., 2010) to constitutive IL-1α and IL-1β expression in the majority of melanoma cell lines independently of disease stage (Qin et al., 2011) and to no IL-1β secretion at all because of lack of expression of one or more inflammasome components (Gehrke et al., 2014). Although these findings do not provide a clear role for IL-1 in isolated melanoma cells in vitro, immunohistochemistry studies imply that IL-1α is uniformly expressed in naevi, primary tumors, and metastases (Qin et al., 2011; Khalili et al., 2012) and, thus, is unrelated to disease progression. In contrast, IL-1β is undetectable in naevi and rarely detected in primary tumors (<10%) but is elevated in metastases (Okamoto et al., 2010; Qin et al., 2011; Khalili et al., 2012; Gehrke et al., 2014). Interestingly, intense IL-1β expression is observed in discrete cells within the tumor, mooted to be melanophages (Gehrke et al., 2014).

A role for host-derived IL-1β, and to a lesser extent IL-1α, in the neovascularization and metastasis of melanoma allografts has been established using recombinant mice (Voronov et al., 2003). Considering that the abundance of macrophages within tumors increases in patients during treatment with MAPK inhibitors and that macrophages can protect melanoma cells from the growth inhibitory effects of MAPK inhibitors (Smith et al., 2014; Wang et al., 2015), we wanted to assess the role of IL-1 signaling in melanoma growth and in the context of MAPK pathway antagonism.

## Results

### IL-1 and IL-1R1 expression is enriched in the tumor stroma

**Figure 1.**
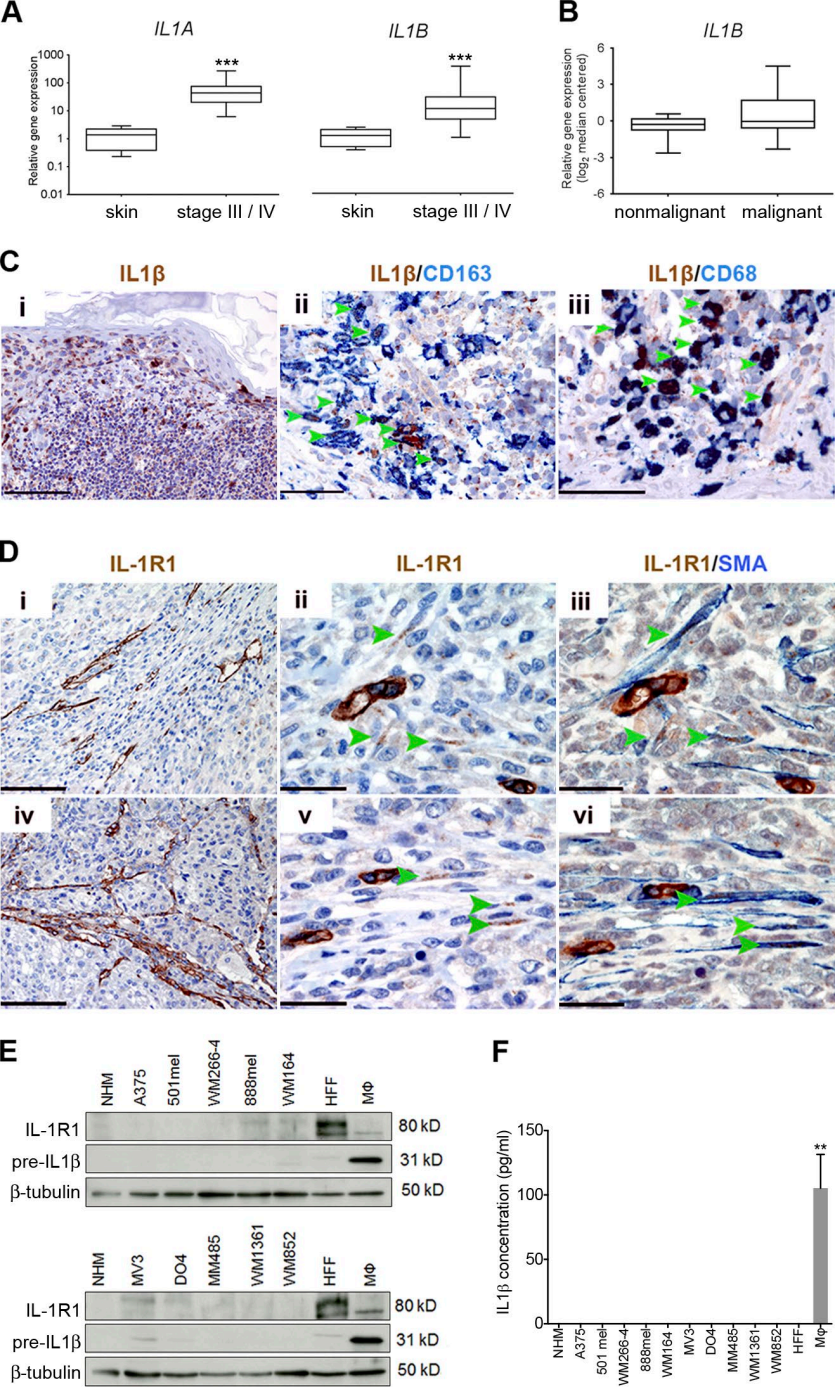
**IL-1 and IL-1R1 expression is enriched in the melanoma stroma.** (A) Real-time qPCR analysis of *IL1A* and *IL1B* expression in stage-III and stage-IV melanoma tumor samples (*n* = 39) relative to expression in human skin samples (*n* = 8). ***, P < 0.001; Mann-Whitney test. (B) Analysis of *IL1B* expression in normal skin and benign nevi samples (nonmalignant; *n* = 25) and cutaneous melanoma samples (malignant; *n* = 45) from an available gene expression dataset (Talantov et al., 2005) accessed through the Oncomine platform. (C) Sections from a case of primary cutaneous melanoma stained for IL-1β, CD163, and CD68 expression as indicated by labels. Bars: (i) 200 µm; (ii) 50 µm; (iii) 33 µm. (D) Serial sections from two skin metastases (i–iii and iv–vi, respectively), stained for IL-1R1 and SMA expression as indicated by the labels. Bars: (i and iv) 200 µm; (ii, iii, v, and vi) 33 µm. (C and D) Arrowheads indicate cells that are clearly double stained. (E) Western blot analysis of IL-1R1 and IL-1β precursor protein expression in a panel of cell lines. Data are representative of three independent experiments. (F) Secreted IL-1β in conditioned media from a panel of melanoma cell lines detected by ELISA. Data are represented as mean ± SEM for three independent samples in each group. **, P < 0.01; Dunn’s multiple comparisons test. (E and F) Macrophages (Mφ) were stimulated with 100 ng/ml IFN-γ and 20 ng/ml LPS.

First, we confirmed the presence of an inflammatory microenvironment in melanoma and detected increased *IL1A* and *IL1B* expression in stage-III and stage-IV melanoma patient samples (Fig. 1 A). Up-regulation of *IL1B* in melanoma was corroborated by microarray data (Talantov et al., 2005) analyzed through the Oncomine platform, demonstrating elevated expression in primary cutaneous melanoma compared with normal skin and benign nevi (Fig. 1 B).

Next, we performed immunohistochemical analysis to assess which cells in the melanoma microenvironment were responsible for the expression of IL-1β. Confirming previous observations (Gehrke et al., 2014), we observed intense staining within discrete cells dispersed throughout tumors (Fig. 1 C, i). Analyzing these specimens for expression of the macrophage markers CD163 and CD68, in combination with IL-1β, revealed that the majority of cells displaying the strongest expression were macrophages (Fig. 1 C, ii and iii, indicated by arrowheads). On average, 81% of CD163^+^ cells infiltrating tumors also stained positive for IL-1β (95% confidence interval = 77–86%; *n* = 6).

To determine which cells within the tumor might be responding to IL-1 stimulation, we performed immunohistochemical analysis for IL-1R1 expression in specimens taken from patient skin metastases. Importantly, IL-1R1 expression was not detectable in melanoma cells. Instead, we observed receptor expression both in endothelial cells (Fig. 1 D) and in fusiform stromal cells (Fig. 1 D, ii and v, indicated by arrowheads), which coexpressed α-smooth muscle actin (α-SMA; Fig. 1 D, iii and vi), revealing them to be melanoma-associated fibroblasts. Thus, stromal cells are the principal candidates responding to IL-1 signaling in melanoma.

To corroborate our findings from immunohistochemical analyses, we analyzed a panel of human melanoma cell lines for the expression of both the IL-1β precursor protein and IL-1R1 by Western blot analysis (further details on the mutational status and origin of the melanoma cells used are outlined in Table S1). In agreement with our findings in melanoma biopsies, we found that established melanoma cell lines express only very low levels of IL-1R1 if any, whereas human foreskin fibroblasts (HFFs) expressed high levels of IL-1R1 (Fig. 1 E). Furthermore, IL-1β precursor protein expression in melanoma cells was negligible (Fig. 1 E), and these cells did not secrete the active, cleaved form of the IL-1β protein (Fig. 1 F). Also, fibroblasts did not express or secrete IL-1β (Fig. 1, E and F). However, as expected, activated macrophages (activated with IFN-γ and LPS) express the precleaved protein (Fig. 1 E) and secrete the active form (Fig. 1 F). This supports the candidacy of macrophages as the primary source of IL-1β in the melanoma microenvironment, in line with our observations in melanoma biopsies. Interestingly, although ordinarily undetectable, we observed IL-1β precursor protein expression in melanoma cells infected with mycoplasma (unpublished data). This suggests that, under normal growth conditions, melanoma cells do not produce significant amounts of precleaved IL-1β, and yet, they have the capacity to do so when stressed.

### Stromal IL-1–IL-1R1 signaling contributes to melanoma growth

**Figure 2.**
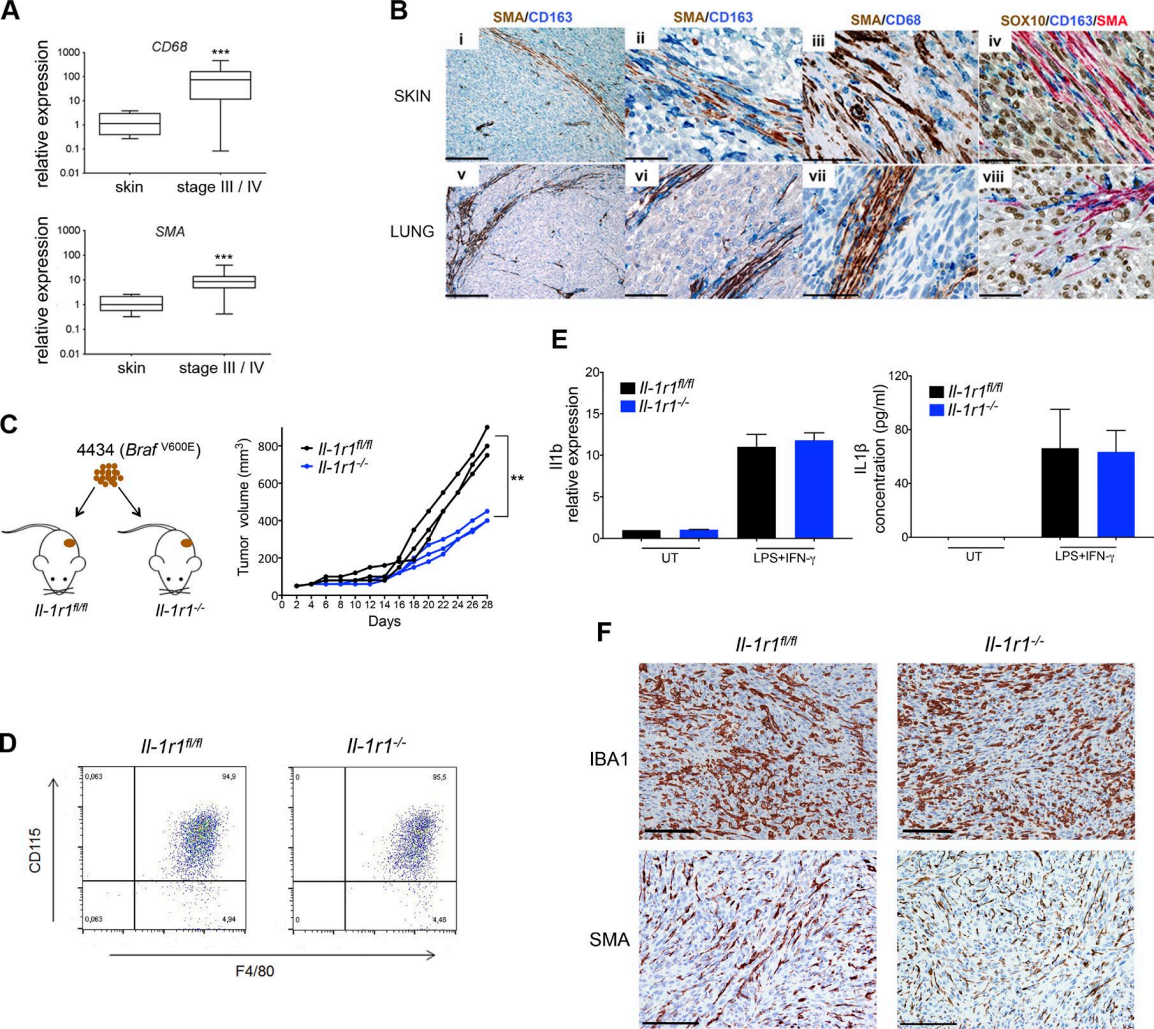
**Macrophages and fibroblasts are organized in the melanoma stroma into inflammatory niches to relay an IL-1 signal that fosters tumor growth.** (A) Real-time qPCR analysis of *CD68* and *SMA* expression in stage-III and stage-IV melanoma tumor samples (*n* = 39) relative to expression in human skin samples (*n* = 8). ***, P < 0.001; Mann-Whitney test. (B) Sections from two cases of skin (i–iv) and lung (v–viii) metastasis of primary cutaneous melanoma, stained for SMA, CD163, CD68, and SOX10 expression as indicated by the labels. Bars: (i and v) 200 µm; (ii, iv, vi, and viii) 50 µm; (iii and vii) 33 µm. (C) Schematic of *Braf^V600E^*-4434 mouse allograft model (left) and growth of individual *Braf^V600E^*-4434 allografts in *Il-1r1^fl/fl^* (*n* = 3) and *Il-1r1^−/−^* (*n* = 3; right) mice. **, P < 0.01; unpaired Student’s *t* test at day 28 after injection. (D) Flow cytometry staining of surface F4/80 and CD115 expression in bone marrow mononuclear cells collected from *Il-1r1^fl/fl^* (left) or *Il-1r1^−/−^* (right) mice and cultured in M-CSF–containing medium for 7 d. Data are representative of three independent experiments. (E) *Il1b* mRNA expression (left) and IL-1β secretion (right) in macrophages generated ex vivo from *Il-1r1^fl/fl^* or *Il-1r1^−/−^* mice stimulated with 100 ng/ml LPS and 50 ng/ml IFN-γ for 24 h, assayed by RT-PCR and ELISA, respectively. Gene expression is shown as fold-change relative to expression in unstimulated macrophages (UT) as mean ± SEM from three independent experiments. ELISA data represent mean ± SEM from two independent experiments. (F) Sections from tumors isolated from *Il-1r1^fl/fl^* and *Il-1r1^−/−^* mice stained for IBA1 and SMA expression as indicated by the labels. Bars, 100 µm. Data are representative of three independent tumors.

In line with the increase in *IL1B* observed in stage-III and stage-IV melanoma (Fig. 1 A) and a role for macrophages as the predominant source and fibroblasts as potential recipients of the cytokine signal, we found an increase in expression of the pan-macrophage marker *CD68* and the cancer-associated fibroblast marker *SMA* in patient melanoma samples compared with normal skin (Fig. 2 A). Moreover, by analyzing melanoma samples for SMA and both CD163 and CD68 expression using immunohistochemistry, we observed fibroblasts and macrophages localized together in bands of connective tissue traversing melanoma metastases taken from skin and lung (Fig. 2 B). Thus, melanoma tumors appear to contain IL-1β–signaling inflammatory niches, a configuration where cross talk between macrophages and stromal cells may be optimized.

To test the importance of IL-1 signaling within the host stroma for melanoma growth, we injected 4434 *Braf^V600E^* melanoma cells, derived from melanoma-bearing *Braf^V600E^* mice (Dhomen et al., 2009), into either syngeneic control mice (*Il-1r1^fl/fl^*) or recently generated *Il-1r1^−/−^* mice that lack both IL-1R1 and the truncated isoform IL-1R3 and thereby display total disruption of IL-1 signaling (Abdulaal et al., 2016). Tumors in *Il-1r1^−/−^* mice grew significantly slower than tumors in control mice, resulting in a profound reduction in tumor size at 28 d after injection (Fig. 2 C). This finding confirms a role for IL-1 signaling in melanoma growth (Voronov et al., 2003) and, furthermore, reveals that a major part of the tumor growth support relies on IL-1 signaling in the host stroma. Monocyte numbers were previously shown to be normal in *Il-1r1^−/−^* mice (Abdulaal et al., 2016), and we now show that bone marrow mononuclear cells derived from *Il-1r1^−/−^* mice can be induced to differentiate ex vivo into macrophages comparable with bone marrow mononuclear cells derived from control mice (Fig. 2 D). We further show that these macrophages both express and secrete levels of IL-1β comparable with macrophages derived from control mice when stimulated with LPS and IFN-γ (Fig. 2 E). Moreover, immunohistochemical analysis to detect IBA1/AIF1 indicated comparable infiltration by macrophages into tumors that grew in *Il-1r1^−/−^* mice as compared with control mice, as immunohistochemical analysis to detect SMA indicated comparable recruitment of fibroblasts (Fig. 2 F). Thus, differences in macrophage and fibroblast recruitment to tumors growing in *Il-1r1^−/−^* compared with control mice is not responsible for the difference observed in tumor growth, implicating a deficiency in stromal IL-1 responsiveness for the reduction in tumor growth.

### Melanoma cells induce IL-1β production by macrophages

**Figure 3.**
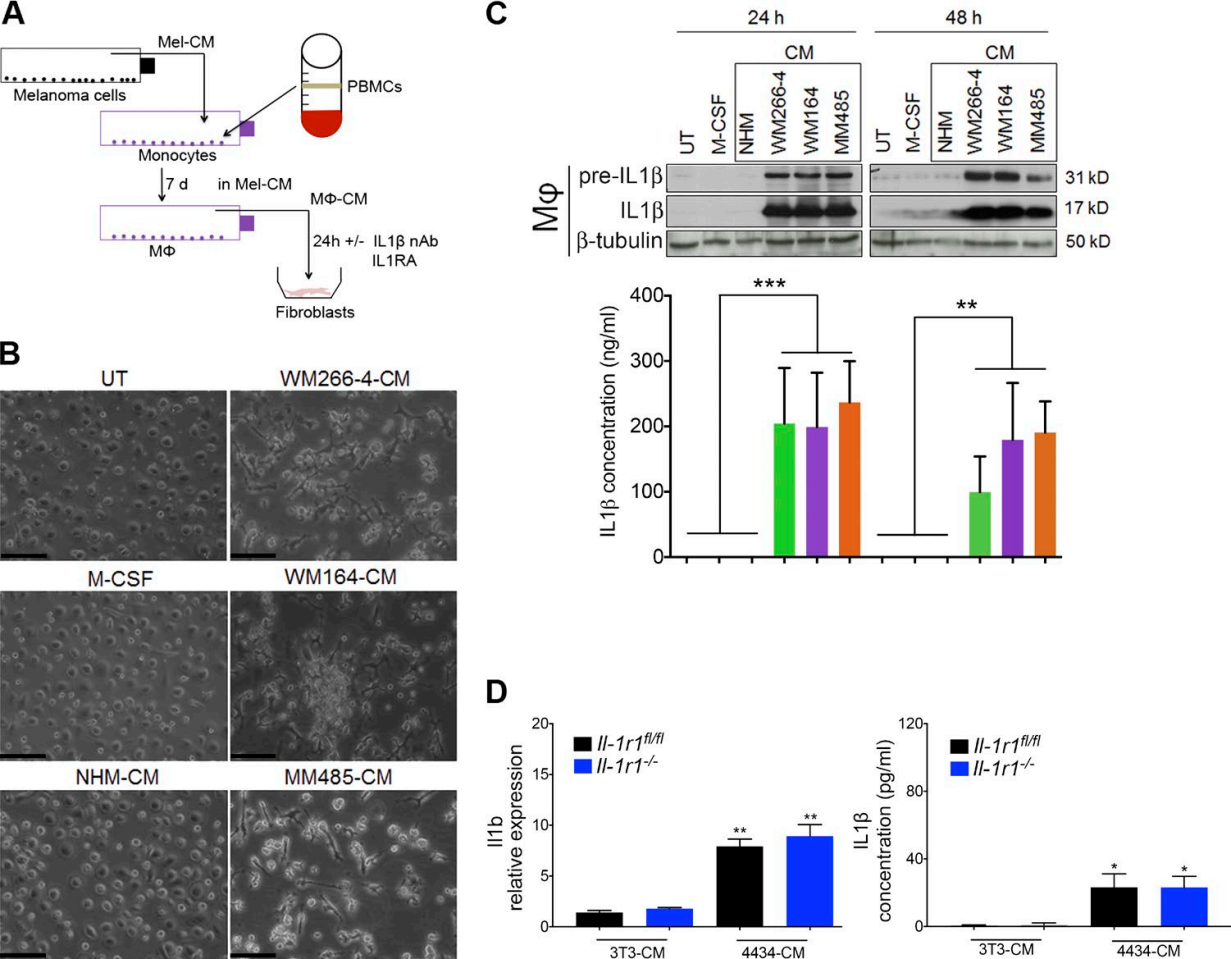
**Melanoma cells initiate an IL-1β signaling cascade that is propagated by macrophages.** (A) Schematic of in vitro co-culture assay of melanoma cells, macrophages (Mφ), and fibroblasts. nAb, neutralizing antibody. (B) Morphology of untreated (UT) macrophages, M-CSF–treated macrophages (M-CSF-Mφ), and macrophages cultured in conditioned media (CM) taken from NHM (NHM-Mφ), WM266-4 (WM266-4–Mφ), WM164 (WM164-Mφ), and MM485 (MM485-Mφ) cells, after 7 d differentiation. Bars,100 µm. Images are representative of three independent experiments. (C, top) Representative Western blot analysis of IL-1β (precursor and mature) protein expression in UT-Mφ, M-CSF–Mφ, NHM-Mφ, WM266-4–Mφ, WM164-Mφ, and MM485-Mφ at 24 and 48 h after differentiation for 7 d. (Bottom) IL-1β secretion in these same macrophages treated at 24 and 48 h after differentiation, detected by ELISA. Data are represented as mean ± SEM from three independent experiments. **, P < 0.01; ***, P < 0.001; Dunn’s multiple comparisons test. Mel-CM–treated samples were compared collectively to controls. (D) *Il1b* mRNA expression (left) and IL-1β secretion (right) in macrophages generated ex vivo from *Il-1r1^fl/fl^* or *Il-1r1^−/−^* mice, stimulated with 3T3-conditioned media or 4434 Mel-CM for 24 h, assayed by RT-PCR and ELISA, respectively. *, P < 0.05; Mann-Whitney test; **, P < 0.01; unpaired Student’s *t* test. Gene expression is shown as fold-change relative to expression in unstimulated macrophages as mean ± SEM from three independent experiments. ELISA data represent mean ± SEM from three independent experiments.

To dissect the cross talk occurring among melanoma cells, macrophages, and fibroblasts in the tumor, we set up an in vitro system using conditioned media from melanoma cells and macrophages (Fig. 3 A). Because we found that melanoma cells do not produce significant amounts of IL-1β themselves (Fig. 1 E) and melanoma cells have previously been shown to stimulate monocyte differentiation into macrophages (Wang et al., 2012), we hypothesized that melanoma cells might trigger IL-1β production and secretion in macrophages. To test this, we cultured human monocytes isolated from peripheral blood in melanoma cell–conditioned medium (Mel-CM) for 7 d (Fig. 3 A). During this time, the morphology of the monocytes became strikingly different to those left untreated, cultured with M-CSF, or cultured in media taken from normal human melanocyte (NHM) cells. In contrast to these control-treated cells that had a typical round fried egg morphology, Mel-CM–treated cells adopted an elongated and dendritic morphology (Fig. 3 B), as described previously (Wang et al., 2012). Moreover, the macrophages displayed high expression of both the precleaved and cleaved IL-1β protein 24 h after the end of the 7-d differentiation phase in Mel-CM (Fig. 3 C). This correlated with high IL-1β secretion by the macrophages, and both the protein expression and secretion were still detectable 48 h after the 7-d differentiation phase (Fig. 3 C). The persistence in IL-1β production even in the absence of Mel-CM suggests that the macrophages may be permanently differentiated. We also found that mouse bone marrow mononuclear cells treated with M-CSF, and thus differentiated into macrophages, also expressed and secreted IL-1β when incubated for a further 24 h in conditioned media from 4434 mouse melanoma cells (Fig. 3 D), whereas cells incubated in conditioned media from untransformed 3T3 cells expressed and secreted low levels of IL-1β (Fig. 3 D). Notably, this phenomenon was consistent in macrophages from both *Il-1r1^fl/fl^* and *Il-1r1^−/−^* mice. These findings point to melanoma cells playing a role in stimulating monocytes to adopt a proinflammatory macrophage phenotype, which results in IL-1β production, among other factors.

### Melanoma cells initiate an IL-1–mediated cross talk between macrophages and fibroblasts that is disrupted by *Il-1r1* ablation

**Figure 4.**
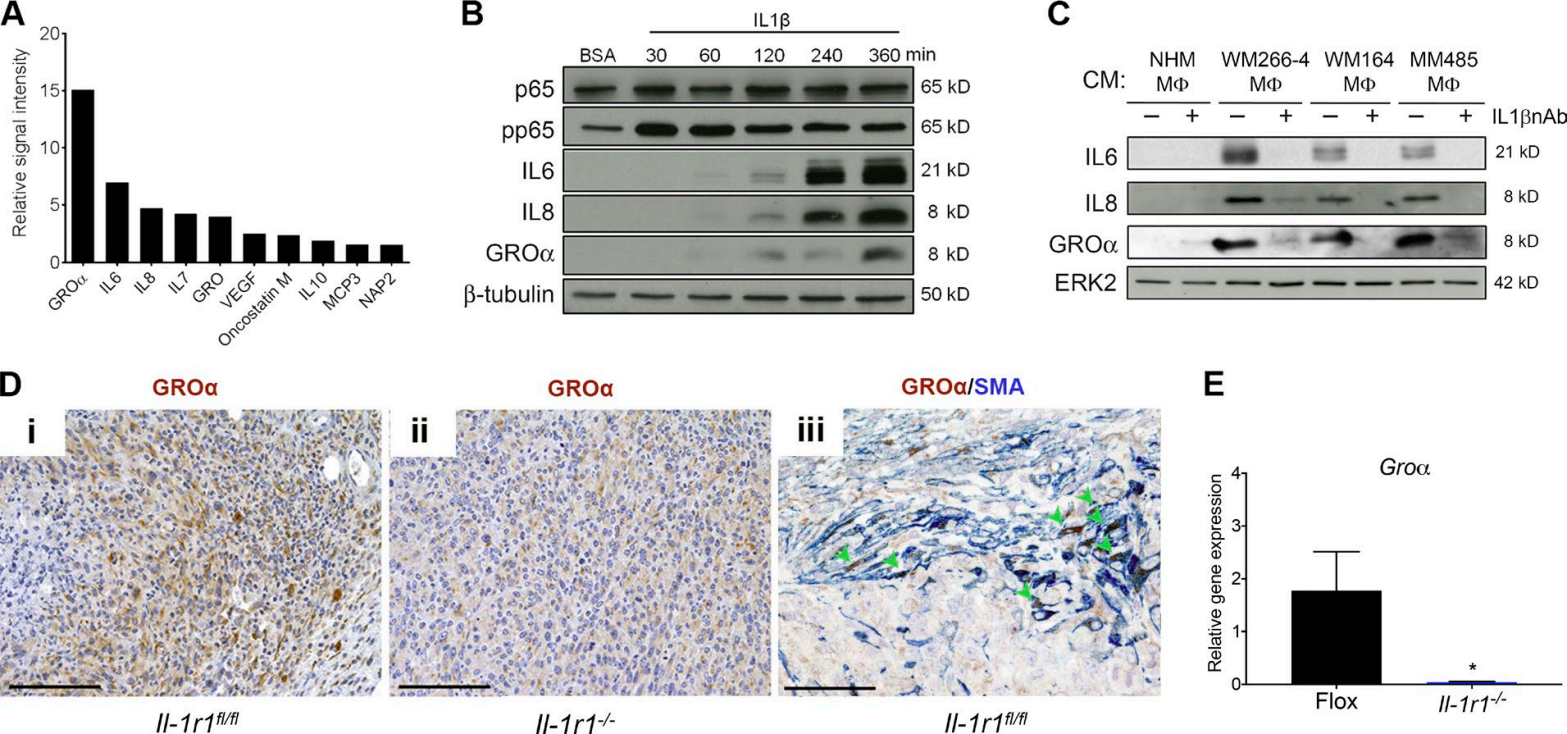
**The IL-1β signaling cascade is further propagated by fibroblasts.** (A) Cytokine array analysis of the normal IMR90 human fibroblast secretome after retroviral transfection with an IL-1A–expressing plasmid. The top ten secreted cytokines are displayed relative to their level in the secretome of normal IMR90 human fibroblasts transfected with control vector. Values represent a mean of two independent experiments. (B) Western blot analysis of p65, pp65, IL-6, IL-8, and GROα expression in HFF cells treated with 100 ng/ml IL-1β for the stated time points. Data are representative of three independent experiments. (C) Western blot analysis of IL-6, IL-8, and GROα expression in HFF cells cultured in conditioned media (CM) taken from NHM macrophage (NHM-Mφ), WM266-4–Mφ, WM164-Mφ, and MM485-Mφ, with 1 µg/ml normal goat IgG control or 1 µg/ml IL-1β neutralizing antibody (IL1βnAb). Data are representative of three independent experiments. (D) Sections from tumors isolated from *Il-1r1^fl/fl^* and *Il-1r1^−/−^* mice stained for GROα and SMA expression as indicated by the labels. (iii) Arrowheads indicate cells that are clearly double stained. Bars: (i and ii)100 µm; (iii) 33 µm. Images are representative of three independent tumors. (E) Real-time qPCR analysis of *Groα* expression in tumors isolated from *Il-1r1^−/−^* mice (*n* = 3) relative to expression in tumors isolated from *Il-1r1^fl/fl^* mice (*n* = 3), at day 28 after injection. Data are represented as mean ± SEM. *, P < 0.05; Mann-Whitney test.

With fibroblasts being the potential recipients of the IL-1 signal in the melanoma microenvironment, we wished to assess the effects of IL-1R1 activation in fibroblasts. For this, we profiled the secretome of fibroblasts stably overexpressing IL-1 using a cytokine antibody array and observed profoundly increased levels of GROα, IL-6, and IL-8 (Fig. 4 A). In line with these data, when we treated human fibroblasts with recombinant IL-1β over a 6-h time course, we observed up-regulation of GROα, IL-6, and IL-8 proteins, accompanied by NF-κB phosphorylation (Fig. 4 B). Because we hypothesized that macrophages trigger IL-1R1 signaling in fibroblasts, we next tested the ability of the Mel-CM–treated macrophages to stimulate these cells (Fig. 3 A). As observed with isolated IL-1β, we found that fibroblasts cultured in conditioned media taken from Mel-CM–differentiated macrophages showed a strong induction in expression of IL-6, IL-8, and GROα (Fig. 4 C). Importantly, this expression was inhibited using an IL-1β–neutralizing antibody, indicating that the induction of IL-6, IL-8, and GROα was dependent on macrophage-derived IL-1β. Of note, macrophages that had been cultured in NHM-conditioned media were not able to stimulate cytokine production in fibroblasts, doubtless because of the lack of IL-1β production (Fig. 3 C and Fig. 4 C).

These data clearly demonstrate that fibroblasts can respond to IL-1β with the production of growth factors such as GROα, and this response would be abolished in the absence of an intact IL-1R1. In line with this, we found that GROα expression was significantly reduced in tumor sections obtained from *Il-1r1^−/−^* mice compared with *Il-1r1^fl/fl^* mice (Fig. 4 D, i and ii). Real-time quantitative PCR (qPCR) analysis to measure *Groα* mRNA expression also confirmed this result (Fig. 4 E). Additionally, GROα was frequently coexpressed in SMA-expressing cells in *Il-1r1^fl/fl^* mice (Fig. 4 D, iii), implicating fibroblasts as the primary source of GROα in melanoma.

### IL-1β expression correlates with IL-8 and GROα expression in melanoma

**Figure 5.**
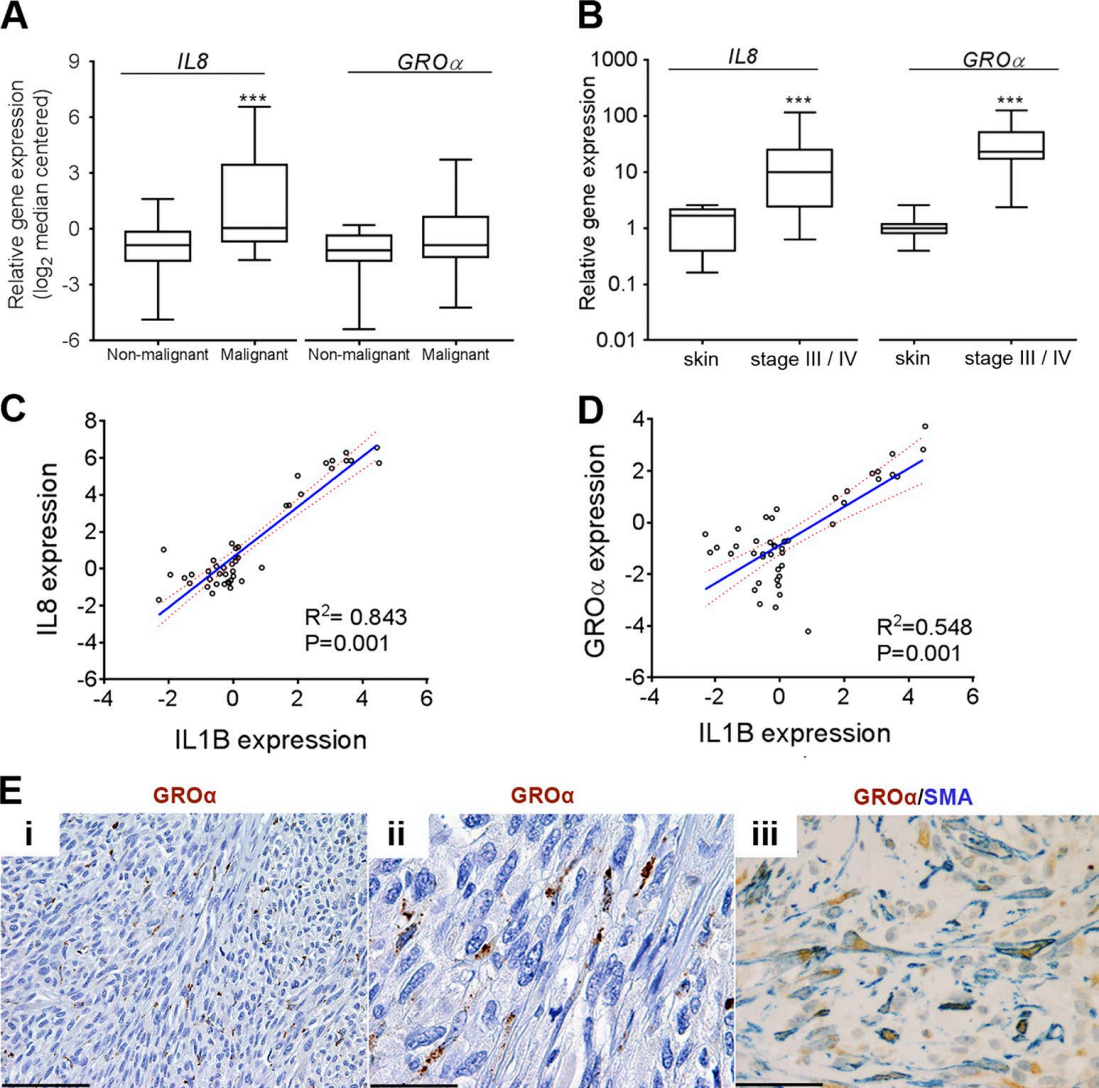
**CXCR2 ligands are up-regulated in human melanomas.** (A) Analysis of *IL8* and *GROα* expression in normal skin and benign nevi samples (nonmalignant; *n* = 25) and cutaneous melanoma samples (malignant; *n* = 45) generated using an available gene expression dataset (Talantov et al., 2005) accessed through the Oncomine platform. (B) Real-time qPCR analysis of *IL8* and *GROα* expression in stage-III and -IV melanoma tumor samples (*n* = 39) relative to expression in human skin samples (*n* = 8). (A and B) ***, P < 0.001; Mann-Whitney test. (C and D) Correlation of *IL8* and *IL1B* (C) and *GROα* and *IL1B* (D) expression in cutaneous melanoma samples (*n* = 45) using an available gene expression dataset (Talantov et al., 2005) accessed through the Oncomine platform. Data are represented as a scatter plot with the regression line (blue) and the 95% confidence interval for the regression line (red dashed lines). (E) Representative sections from skin metastases of primary cutaneous melanoma stained for GROα and SMA expression as indicated by the labels. Bars: (i) 100 µm; (ii and iii) 33 µm.

Altogether, these data provide evidence for a relay of signals among melanoma cells, macrophages, and fibroblasts in the melanoma microenvironment. As IL-6, IL-8, and GROα have already been demonstrated to be important cytokines for melanoma growth and progression (Schadendorf et al., 1993; Bar-Eli, 1999; Haghnegahdar et al., 2000; Huang et al., 2002; von Felbert et al., 2005; Varney et al., 2006), this provides a link between IL-1 signaling in the stroma and melanoma growth support. In line with this, we observed a marked increase in *IL8* and *GROα* mRNA expression in human melanoma samples (Fig. 5, A and B). The expression of both *IL8* and *GROα* in primary melanomas correlated strongly with *IL1B* expression (Fig. 5, C and D), consistent with their expression being largely dependent on *IL1B* expression. Immunohistochemical analysis of GROα expression in specimens taken from patient skin metastases revealed that fibroblasts are one of the major producers of GROα in melanoma (Fig. 5 E, i–iii), substantiating earlier observations in mouse tumors (Fig. 4 D).

### IL-1β–activated fibroblasts confer tolerance to BRAF/MEK combination therapy through NF-κB and BCL2

**Figure 6.**
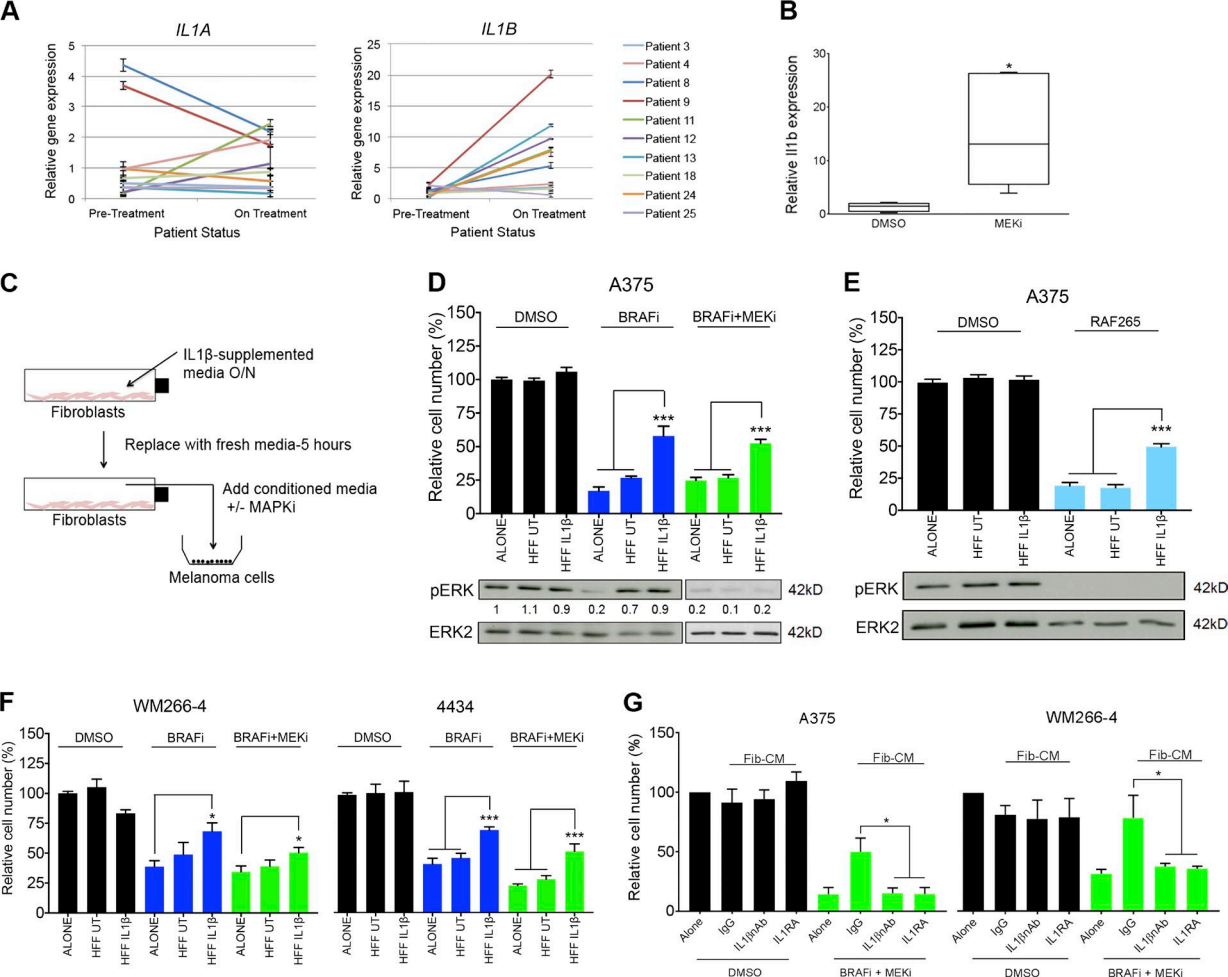
**The IL-1β signaling cascade is augmented by and confers tolerance to MAPK pathway inhibitors.** (A) Real-time qPCR analysis of *IL1A* (left) and *IL1B* (right) expression in tumors from *BRAF^V600E^*-positive metastatic melanoma patients undergoing treatment with BRAFi alone or a BRAFi and MEKi combination (*n* = 10). Each line represents relative gene expression in an individual patient pretreatment and at 10–14 d on treatment, with error bars representing mean ± SD from three repeats. (B) Real-time qPCR analysis of *Il1b* expression in *Braf^V600E^*-4434 allografts from C57J/B6 mice treated with 25 mg/kg/d PD184352 (MEKi; *n* = 5) or vehicle (DMSO; *n* = 5) for 20 d. Unpaired Student’s *t* test was used. (C) Schematic of in vitro co-culture assay of melanoma cells and fibroblasts using conditioned media from IL-1β–stimulated fibroblasts in combination with MAPK signaling inhibitors (MAPKi). O/N, overnight. (D, top) Growth assay of A375 cells treated with 1% DMSO, 1 µM PLX4032 (BRAFi), or 0.5 µM both PLX4032 and selumetinib (MEKi), cultured in nonconditioned media or conditioned media taken from unstimulated fibroblasts or fibroblasts previously stimulated with IL-1β. UT, untreated. (Bottom) Representative Western blot analysis and quantification of pERK expression in A375 cells treated as just described, for 24 h. (E, top). Growth assay of A375 cells treated with 1% DMSO or 1 µM RAF265 (pan-RAFi), cultured in conditioned media as in D. (Bottom) Representative Western blot analysis of pERK expression in A375 cells treated as just described for 24 h. (D and E) Western blot data are representative of two independent experiments. (F) Growth assay of WM266-4 (left) and 4434 (right) cells treated as in D. (G) Growth assay of A375 (left) and WM266-4 (right) cells treated with 1% DMSO or 0.5 µM both PLX4032 and selumetinib, cultured in nonconditioned media or conditioned media taken from fibroblasts previously cultured in media taken from Mel-CM–treated macrophages supplemented with 1 µg/ml normal goat IgG control, 1 µg/ml IL-1β neutralizing antibody (IL1βnAb), or 1 µg/ml IL-1RA. (D–F) Data are represented as mean ± SEM from at least three independent experiments with a minimum of eight repeats. Tukey’s multiple comparisons test was used. (G) Data are represented as mean ± SEM from at least three independent experiments. Mann-Whitney test was used. For all growth assays, cells were treated for 48 h, and cell number was assayed by crystal violet staining. *, P < 0.05; ***, P < 0.001.

As mentioned earlier, we had previously detected increased macrophage abundance in *BRAF^V600E^*-positive melanomas from patients that had been treated with BRAF and MEKi for 10–14 d (Smith et al., 2014). As we had identified macrophages as a crucial source of IL-1–induced growth support signals, we wanted to analyze IL-1 expression in these patient samples (for further patient details, see Table S2). This analysis revealed a decrease in *IL1A* expression in patients on treatment compared with pretreatment (Fig. 6 A, left), consistent with previous observations and with *IL1A* being a MAPK signaling target (Khalili et al., 2012). However, we detected a clear increase in *IL1B* expression in the majority of samples from patients on treatment compared with pretreatment (Fig. 6 A, right), consistent with our previous finding of increased macrophage abundance in patient tumors on treatment (Smith et al., 2014). Real-time qPCR analysis also demonstrated increased *Il1b* mRNA in 4434-derived mouse allograft tumors treated with MEKi (Fig. 6 B).

The increase in macrophage abundance and *IL1B* expression on treatment could contribute to the adaptive response of melanoma cells that promotes treatment tolerance (Smith and Wellbrock, 2016). However, in contrast to TNF, which has been shown to directly prevent melanoma cell death in the presence of BRAF signaling inhibition, IL-1β cannot prevent cell death when BRAF signaling is inhibited (Gray-Schopfer et al., 2007; Smith et al., 2014). Nonetheless, because macrophage-derived IL-1β can activate fibroblasts to produce cytokines that could hypothetically protect against MAPK inhibitors, we subsequently examined the ability of melanoma cells exposed to fibroblast-conditioned media (Fib-CM) pretreated with IL-1β (IL-1β–Fib-CM; Fig. 6 C) to tolerate the BRAFi vemurafenib, the pan-RAF inhibitor RAF265, the MEKi selumetinib, or, indeed, a combination of these therapeutics. In line with previously published work and a role for secreted factors in ERK reactivation upstream of MEK (Straussman et al., 2012), we found that A375 cells cultured in medium from unstimulated fibroblasts were protected to an extent against BRAF inhibition, but the factors present in the medium were not sufficient to protect from a combination of BRAF and MEKi treatment (Fig. 6 D). However, A375 cells cultured in IL-1β–Fib-CM were protected not only from BRAF inhibition, but also from BRAF/MEKi combination (Fig. 6 D). Moreover, IL-1β–Fib-CM also protected A375 cells from pan-RAF inhibition (Fig. 6 E). Similar effects were observed in WM266-4 and 4434 melanoma cell lines (Fig. 6 F).

BRAF inhibition resulted in loss of ERK phosphorylation, but this was rescued when cells were cultured in media taken from either unstimulated fibroblasts or IL-1β–activated fibroblasts (Fig. 6 D), as previously described (Straussman et al., 2012). However, ERK reactivation was not observed when melanoma cells were treated with BRAF/MEKi combination therapy (Fig. 6 D) and, similarly, when treated with a pan-RAF inhibitor (Fig. 6 E). Thus, our data confirm that fibroblasts can protect melanoma cells from BRAF inhibition through reactivation of the MAPK pathway. However, we demonstrate that, when activated by IL-1β, fibroblasts can protect melanoma cells from MEK inhibition through an ERK-independent mechanism.

To more closely model heterotypic cell interactions in the tumor microenvironment, we cultured A375 and WM266-4 melanoma cells in media taken from fibroblasts that had themselves previously been cultured in conditioned media taken from Mel-CM–differentiated macrophages. We found that the melanoma cells were indeed protected against BRAF and MEK inhibition (Fig. 6 G), although protection was lost if macrophage-conditioned medium was preincubated with IL-1β–neutralizing antibody or IL-1 receptor antagonist (IL-1RA; Fig. 6 G), further confirming a role for macrophage secretion of IL-1β in protecting melanoma cells against MAPK inhibitors.

**Figure 7.**
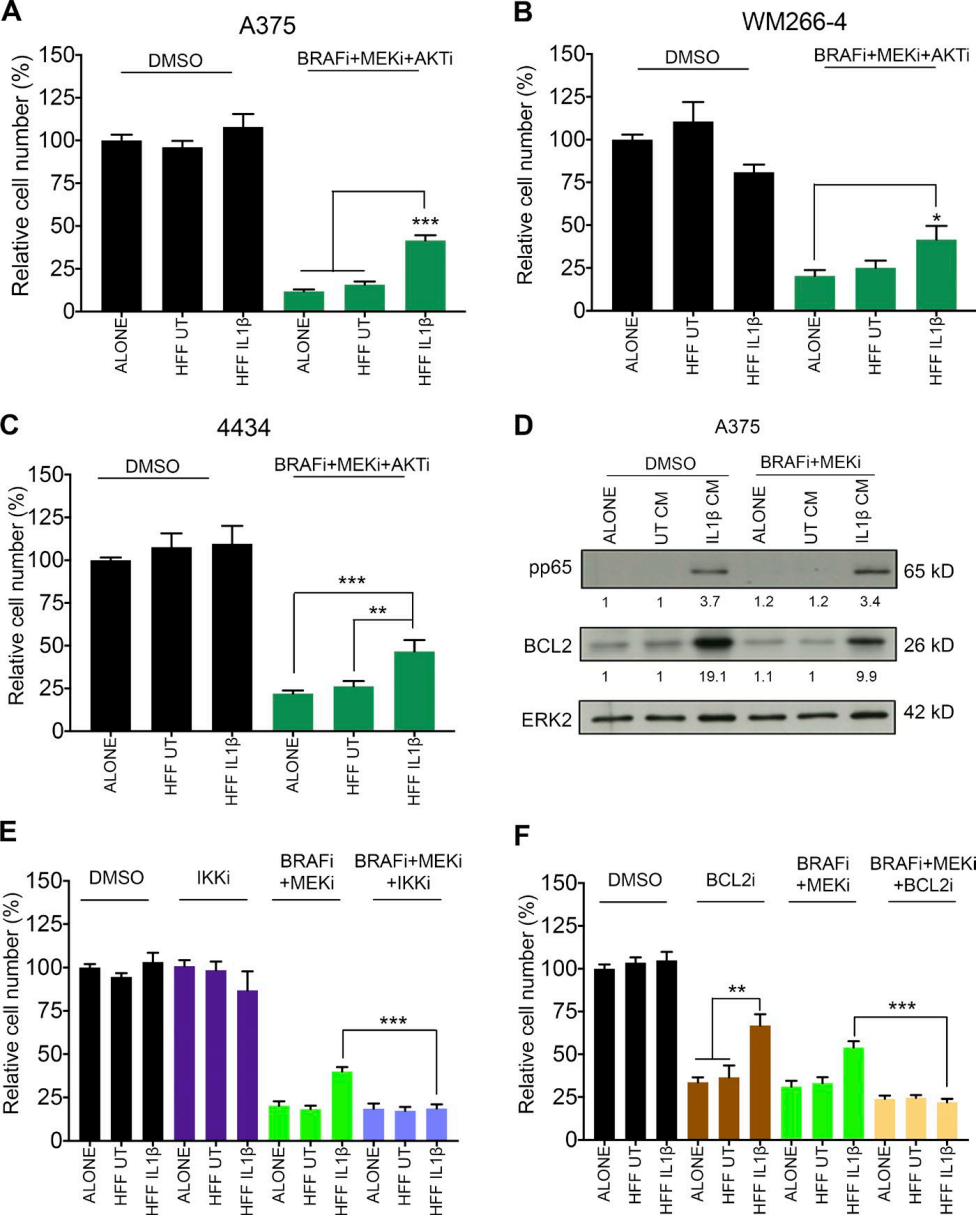
**IL1-β–activated fibroblasts mediate tolerance to BRAF/MEK combination therapy through NF-κB and BCL2.** (A) Growth assay of A375 treated with 1% DMSO or 0.5 µM both PLX4032 and selumetinib and 1 µM MK-2206 (AKTi), cultured in nonconditioned media or conditioned media taken from unstimulated fibroblasts or fibroblasts previously stimulated with IL-1β. UT, untreated. (B) Growth assay of WM266-4 (B) and 4434 (C) cells treated as in A. (A–C) *, P < 0.05; **, P < 0.01; ***, P < 0.001; Tukey’s multiple comparisons test. (D) Representative Western blot analysis and quantification of pp65 and BCL2 expression in A375 cells treated with 1% DMSO or 0.5 µM both PLX4032 and selumetinib cultured in conditioned media (CM) as in A for 24 h. Data are representative of two independent experiments. (E) Growth assay of A375 cells treated with 1% DMSO, 0.2 µM Bay 11-7082 (IKKi), 0.5 µM both PLX4032 and selumetinib or 0.5 µM both PLX4032 and selumetinib, and 0.2 µM Bay 11-7082, cultured in conditioned media as in A. (F) Growth assay of A375 cells as in E but with 0.2 µM obatoclax (BCL2i) instead. (E and F) **, P < 0.01; Tukey’s multiple comparisons test; ***, P < 0.001; unpaired Student’s *t* test. (A–C, E, and F) Data are represented as mean ± SEM from at least three independent experiments with a minimum of seven repeats. For all growth assays, cells were treated for 48 h, and cell number was assayed by crystal violet staining.

We further confirmed that IL-1β–Fib-CM–induced tolerance to MAPK antagonism is not PI3K dependent, as IL-1β–Fib-CM also protected A375 cells from a BRAF/MEK/AKT inhibitor combination (Fig. 7 A). This effect was also observed in WM266-4 cells (Fig. 7 B) and 4434 cells (Fig. 7 C). Next, we analyzed how IL-1β–Fib-CM enables melanoma cells to overcome MAPK inhibition in an ERK-independent manner. We found that NF-κB p65 phosphorylation and BCL2 expression were increased in melanoma cells treated with IL-1β–Fib-CM (Fig. 7 D). Importantly, this was not affected by BRAF/MEKi combination treatment (Fig. 7 D). The fact that an IκB kinase inhibitor (Fig. 7 E) or a BCL2 inhibitor (Fig. 7 F) could overcome the protective effect conferred by IL-1β–Fib-CM suggests that NF-κB activation and BCL2 up-regulation contribute to the survival signals.

### IL-1β–activated fibroblasts protect melanoma cells from MAPK inhibition by signaling through the CXCR2 receptor

**Figure 8.**
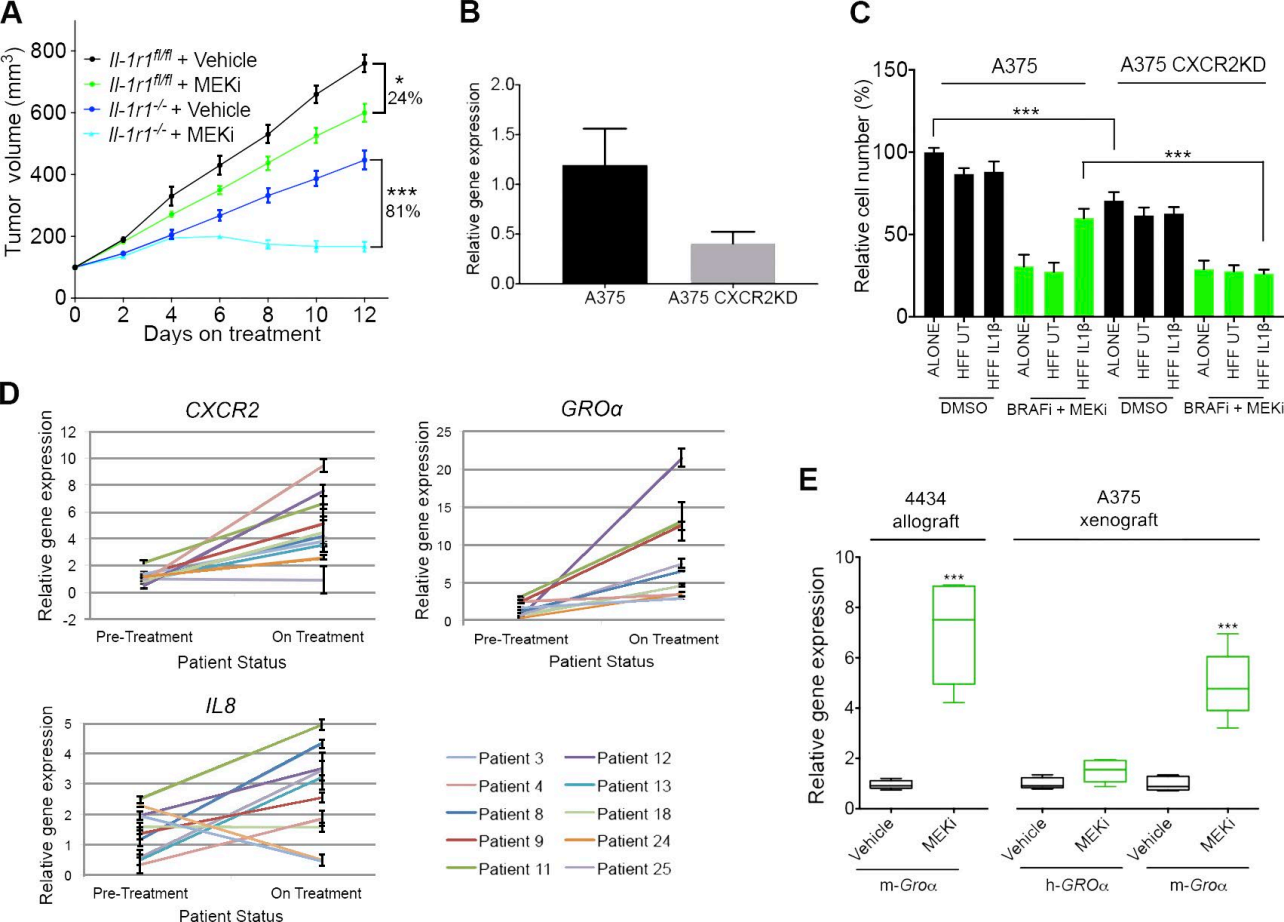
**CXCR2 signaling confers tolerance to MAPK pathway inhibitors.** (A) Growth of *Braf^V600E^*-4434 allografts in vehicle-treated *Il-1r1^fl/fl^* mice (*n* = 5), *Il-1r1^fl/fl^* mice treated with 25 mg/kg PD184352 (MEKi; *n* = 5), vehicle-treated *Il-1r1^−/−^* mice (*n* = 6), and *Il-1r1^−/−^* mice treated with 25 mg/kg PD184352 (*n* = 4). Data are represented as mean ± SEM. *, P < 0.05; ***, P < 0.001; Tukey’s multiple comparisons test at day 12 on treatment. (B) Real-time qPCR analysis of *CXCR2* expression in A375 CXCR2KD cells relative to expression in A375 cells (*n* = 4). Data are represented as mean ± SEM. (C) Growth assay of A375 and A375 CXCR2KD cells treated with 1% DMSO or a combination of 0.5 µM both PLX4032 and selumetinib, cultured in nonconditioned media or conditioned media taken from unstimulated fibroblasts or fibroblasts previously stimulated with IL-1β for 48 h, detected by crystal violet staining. Data are represented as mean ± SEM from three independent experiments with nine repeats. ***, P < 0.05; unpaired Student’s *t* test. UT, untreated. (D) Real-time qPCR analysis of *CXCR2*, *GROα*, and *IL8* expression in tumors from *BRAF^V600E^*-positive metastatic melanoma patients undergoing treatment with BRAFi alone or a BRAFi and MEKi combination (*n* = 10). Each line represents relative gene expression in an individual patient pretreatment and at 10–14 d on treatment, with error bars representing mean ± SD from three repeats. (E) Real-time qPCR analysis of *Groα* expression in *Braf^V600E^*-4434 allografts from C57J/B6 mice treated with 25 mg/kg/d PD184352 (*n* = 5) or vehicle (*n* = 5) for 20 d (left) and both human *GROα* (h-*GROα*) and mouse *Groα* (m-*Groα*) expression in A375 human melanoma xenografts implanted in nude mice treated with 10 mg/kg/d AZD6244 (MEKi; *n* = 5) or vehicle (*n* = 5) for 30 d (right). ***, P < 0.05; unpaired Student’s *t* test.

To test the importance of IL-1β–mediated stromal signals in conferring tolerance to MAPK inhibition in vivo, we again injected 4434 into *Il-1r1^fl/fl^* and *Il-1r1^−/−^* mice and analyzed tumor growth in the presence of MEKi. Whereas MEK inhibition in control mice resulted in ∼24% reduction in tumor growth, ∼81% reduction was observed in *Il-1r1^−/−^* mice treated with MEKi (Fig. 8 A), clearly demonstrating that stromal IL-1 signals are important in promoting tolerance to MAPK inhibition in melanoma.

Next, we wished to dissect the stromal signaling that IL-1 induces to promote MAPK inhibitor tolerance. As we have shown that IL-1β stimulates IL-6, IL-8, and GROα production in fibroblasts, these cytokines could potentially contribute to the stromal-derived tolerance. However, we found that IL-6 induced growth inhibition in melanoma cells (unpublished data) and was therefore deemed an unlikely candidate. GROα and IL-8 are both ligands for the CXCR2 receptor, so to assess whether CXCR2 plays a role in the inflammatory niche–mediated tolerance, we used A375 cells in which receptor expression is depleted by expression of a CXCR2 targeting shRNA (A375 CXCR2 knockdown [CXCR2KD] cells; Fig. 8 B). Whereas IL-1β–Fib-CM offered significant protection against BRAF/MEK combination treatment in A375 cells, in A375 CXCR2KD cells, this protection was lost (Fig. 8 C). A375 CXCR2KD cells grew at a slightly slower rate than A375 cells (Fig. 8 C), which could be linked to a basal growth-promoting role of CXCR2 signaling (Schadendorf et al., 1993; Singh et al., 1994; Haghnegahdar et al., 2000).

Given the potential role for CXCR2 in the inflammatory niche–mediated tolerance and because *IL1B* expression was up-regulated in tumor biopsies from patients after 10–14 d of treatment with BRAF and MEKi’s, we analyzed these tumors for *CXCR2* and its ligands *GROα* and *IL8*. We observed an increase in *CXCR2* and *GROα* expression (Fig. 8 D). However, we only found a slight increase in *IL8* expression and, in several cases, even a reduction in *IL8* expression in patients on treatment (Fig. 8 D), which confirms previous observations (Sanmamed et al., 2014; Wilmott et al., 2014). This renders IL-8 an unlikely candidate for the stimulation of CXCR2 in the presence of MAPK antagonists. Furthermore, qPCR analysis revealed increased *Groα* mRNA in MEKi-treated 4434 allografts compared with vehicle-treated controls (Fig. 8 E, left), and specifically, mouse *Groα* mRNA and not human *GROα* mRNA was up-regulated in MEKi-treated A375 xenografts (Fig. 8 E, right; Smith et al., 2013), confirming the tumor stroma as the source of GROα.

**Figure 9.**
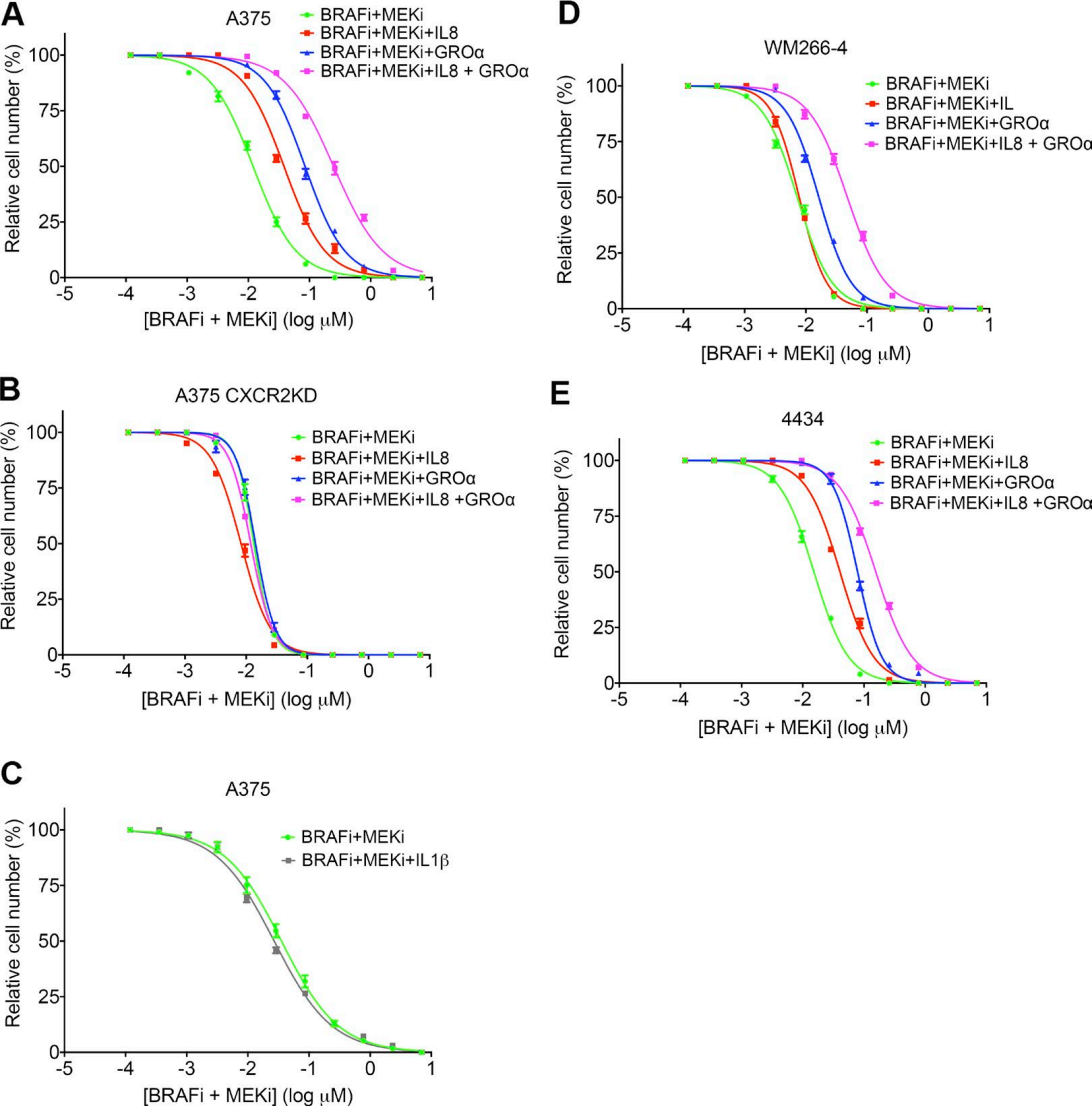
**CXCR2 ligands protect melanoma cells from BRAF and MEK inhibition.** (A and B) Drug dose–response analysis of A375 (A) and A375 CXCR2KD (B) cell survival in response to BRAFi and MEKi, in combination with 100 ng/ml IL-8, 100 ng/ml GROα, or 100 ng/ml IL-8 and GROα for 72 h, detected by crystal violet staining. (C) Drug dose–response analysis of A375 cell survival in response to BRAFi and MEKi, in combination with 100 ng/ml IL-1β for 72 h, detected by crystal violet staining. (D and E) Drug dose–response analysis of WM266-4 (D) and 4434 (E) cell survival in response to BRAFi and MEKi as in A. Data are represented as mean ± SEM from two independent experiments where each treatment was performed on samples in triplicate.

In line with these findings, GROα and IL-8 were able to protect melanoma cells from BRAFi- and MEKi-induced death. Addition of IL-8, GROα, or a combination of both in the presence of BRAFi and MEKi increased the 50% effective concentration by approximately fourfold (from 0.01 to 0.04 µM), ninefold (from 0.01 to 0.09 µM), and 25-fold (from 0.01 to 0.25 µM), respectively, in A375 cells (Fig. 9 A). Therefore, GROα conferred more protection than IL-8, but the combination of the two cytokines offered the best protection, confirming our finding that GROα is the more likely candidate for the stimulation of CXCR2. This protective effect was lost in A375 CXCR2KD cells (Fig. 9 B). As anticipated, treatment with IL-1β alone did not confer any protection from BRAF/MEKi-induced cell death in A375 cells (Fig. 9 C). GROα, IL-8, and a combination of GROα and IL-8 also protected WM266-4 and 4434 cells with similar effect (Fig. 9, D and E), although IL-8 did not appear to offer any protection to WM266-4 cells.

### CXCR2 inhibition synergizes with MEK inhibition in vivo to significantly reduce tumor growth

**Figure 10.**
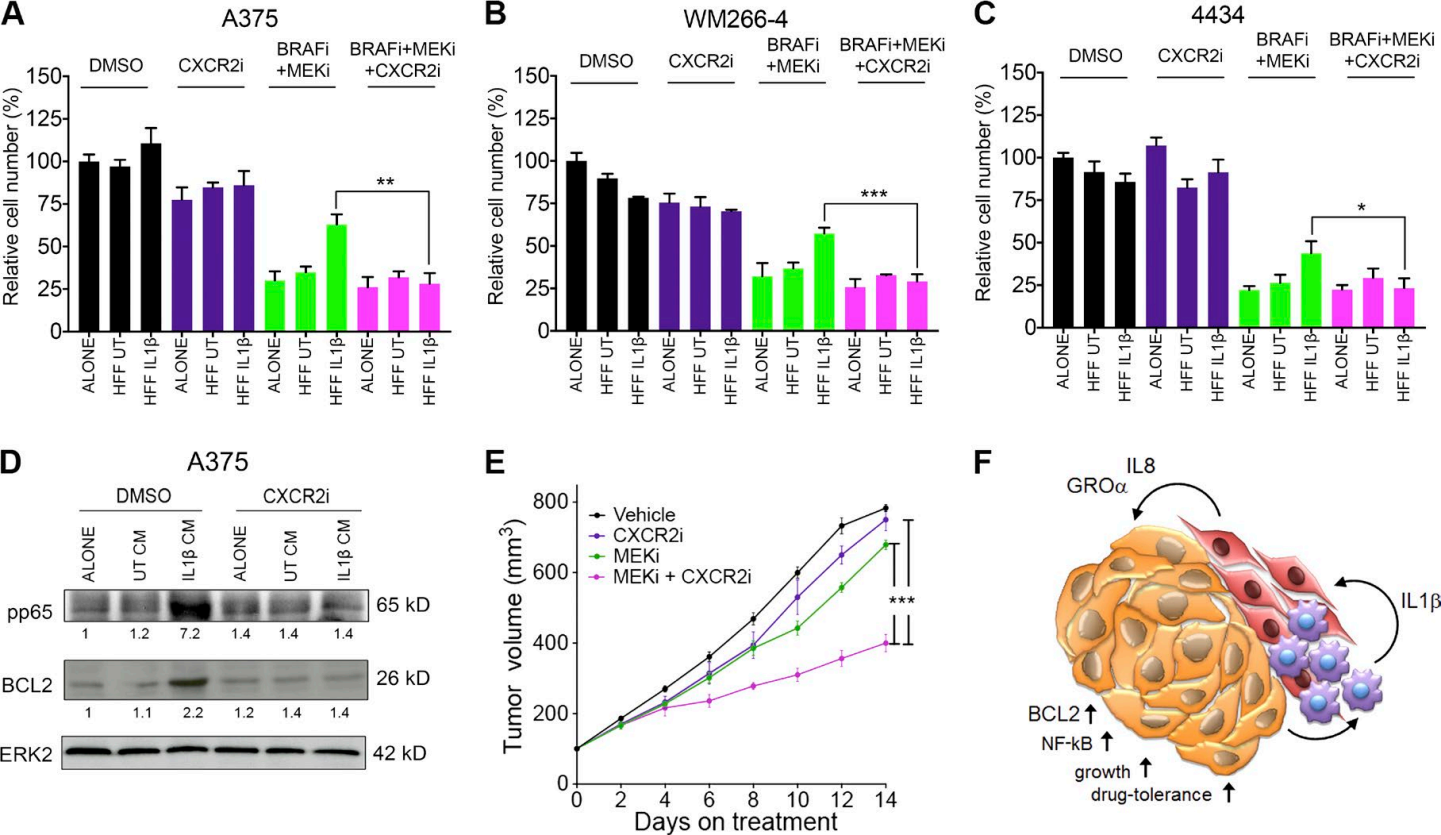
**CXCR2i and MEKi synergizes in vivo to effectively reduce tumor growth.** (A–C) Growth assay of A375 (A), WM266-4 (B), and 4434 (C) cells treated with 1% DMSO, 0.5 µM SB225002 (CXCR2i), 0.5 µM both PLX4032 and selumetinib (BRAFi + MEKi), or 0.5 µM BRAFi, MEKi, and CXCR2i, cultured in nonconditioned media or conditioned media taken from unstimulated fibroblasts or fibroblasts previously stimulated with IL-1β for 48 h, detected by crystal violet staining. Data are represented as mean ± SEM from three independent experiments (A) or two independent experiments (B and C), with a minimum of five repeats. *, P < 0.05; **, P < 0.01; ***, P < 0.001; unpaired Student’s *t* test. UT, untreated. (D) Representative Western blot analysis and quantification of pp65 and BCL2 expression in A375 cells treated with 1% DMSO or 0.5 µM SB225002 (CXCR2i) cultured in conditioned media (CM) as in A for 24 h. (E) Growth of *Braf^V600E^*-4434 allografts in vehicle-treated C57J/B6 mice (*n* = 8), mice treated with 25 mg/kg PD184352 (MEKi; *n* = 8), mice treated with 30 mg/kg sch-527123 (CXCR2i; *n* = 5), and mice treated with 25 mg/kg PD184352 and 30 mg/kg sch-527123 (*n* = 5). Data are represented as mean ± SEM. ***, P < 0.001; Tukey’s multiple comparisons test at day 14 on treatment. (F) Model of cross talk among melanoma cells, macrophages, and fibroblasts located in inflammatory niches in melanoma tumors, which leads to survival in the presence of MAPK signaling inhibitors.

Our data emphasize that IL-1β cannot directly induce tolerance to MAPK inhibition in melanoma cells but requires signaling through CXCR2. Furthermore, we found that *CXCR2* expression is up-regulated in the majority of tumors in patients on treatment with MAPK inhibitors (Fig. 8 D). Thus, pharmacologically inhibiting CXCR2 signaling represents an attractive therapeutic approach that would prevent IL-1β–activated fibroblasts from protecting melanoma cells from MAPK inhibition. Indeed, using the potent and highly selective CXCR2 inhibitor SB225002 (Bento et al., 2008; Manjavachi et al., 2010) resulted in a significant loss of IL-1β–Fib-CM–mediated protection from BRAF/MEKi combination treatment in A375 (Fig. 10 A), WM266-4 (Fig. 10 B), and also 4434 (Fig. 10 C) cells. In line with this, CXCR2 inhibition blocked the IL-1β–Fib-CM–induced p65 phosphorylation and BCL2 up-regulation (Fig. 10 D). Together, these data suggest that interfering with CXCR2 signaling could be very effective in improving responses to MAPK inhibitor therapy.

Thus, to test the effect of CXCR2 inhibition in vivo, we again used the 4434 allograft melanoma model. We treated 4434 allograft–bearing mice with an MEKi alone or in combination with the CXCR2 inhibitor sch-527123 (navarixin), which has been optimized for clinical use (Holz et al., 2010; Nair et al., 2012). A significant reduction in tumor growth was observed in mice treated with the combination of navarixin and MEKi compared with either treatment alone (Fig. 10 E). This suggests that targeting CXCR2 in combination with MAPK signaling could improve initial responses to MAPK inhibitors in melanoma patients.

## Discussion

The biggest hurdle yet to be overcome for the treatment of disseminated melanoma using targeted therapies is the emergence of resistant disease. It is clear from our data and previous studies (Straussman et al., 2012; Smith et al., 2014; Hirata et al., 2015; Wang et al., 2015) that innate immune cells and stromal cells of the melanoma microenvironment play a role in this process in allowing melanoma cells to rapidly tolerate MAPK signaling inhibition before genetic mechanisms of resistance are acquired. We show that macrophages and fibroblasts are located in inflammatory niches in melanomas and are responsible for elevated IL-1 signaling in the melanoma stroma. We show that cross talk between melanoma cells, macrophages, and fibroblasts initiates an IL-1 signaling cascade that generates a CXCR2-stimulating secretome, which ultimately leads to enhanced melanoma cell survival in the presence of MAPK signaling inhibition, via BCL2 up-regulation (Fig. 10 F). We show that blocking IL-1R1 signaling or CXCR2 signaling synergizes effectively with MEK inhibition in vivo, suggesting this as a means to delay the onset of resistance that presently too frequently occurs in melanoma patients.

Monocyte differentiation into macrophages is regulated by several cytokines, including but not limited to M-CSF/CSF-1 (Wang et al., 2012). Typically, production and release of IL-1β by macrophages requires activation of NF-κB by cytokine or Toll-like receptor signaling to induce gene expression and, subsequently, activation of the inflammasomes by pathogen-associated or damage-associated molecular patterns to stimulate secretion (Garlanda et al., 2013). Analysis of the secretome of melanoma cells has revealed several soluble factors including cytokines such as M-CSF/CSF-1, CCL2, IFN-γ, IL-6, GM-CSF, leukemia inhibitory factor, and vascular endothelial growth factor A, as well as ligands for Toll-like receptors such as high mobility group box proteins and heat shock proteins, which could participate in driving the differentiation of monocytes to macrophages and/or stimulate IL-1β expression (unpublished data; Ohanna et al., 2011; Wang et al., 2012; Obenauf et al., 2015), whereas reactive oxygen species generated by metabolically active melanoma cells or damage-associated molecular patterns released by dying melanoma cells could all potentially activate the inflammasomes of macrophages. The action of the various factors together in a single secretome is very complex, and indeed, they play a redundant role in inducing monocyte differentiation; moreover, it appears the expression of individual factors is highly heterogeneous among individual melanoma cell lines (Wang et al., 2012). Therefore, trying to block IL-1β production therapeutically would be less practicable than attempting to block IL-1 action by neutralizing antibody or receptor antagonist, which we show antagonize the protective effect conveyed by macrophages against MAPK antagonism (Fig. 6 G).

It is known from previous studies that the cells of the tumor microenvironment are themselves influenced by MAPK signaling inhibition (Hirata et al., 2015; Wang et al., 2015). We have previously observed a marked increase in the number of macrophages in human tumor biopsies from patients on treatment with vemurafenib or a combination of dabrafenib and trametinib, compared with pretreatment (Smith et al., 2014). This may potentially explain the increase in IL-1 signaling we also observed in the tumor biopsies from patients on treatment compared with pretreatment. Therefore, macrophages may be recruited to melanomas upon MAPK inhibitor treatment, which allows for a relatively quick development of drug tolerance through IL-1 signaling activation and subsequent stimulation of fibroblasts. This suggests that targeting this mechanism in combination with MAPK inhibitor therapy may result in a much more potent response in patients.

Previous work has demonstrated the importance of CXCR2 signaling for growth in mouse transplantation melanoma models (Singh et al., 2009a,b) and other cancer models (Tazzyman et al., 2011). To our knowledge, however, CXCR2 signaling has not been previously implicated in promoting tolerance to MAPK signaling inhibition in melanoma; thus, we describe a novel mechanism by which cells can tolerate MAPK therapy. IL-8 has been shown in vitro to stimulate neuroblastoma RAS viral oncogene homolog–mutant melanoma cell invasion in the presence of a BRAFi through paradoxical activation of the MAPK pathway. Yet, this was overcome by inhibiting MEK (Sanchez-Laorden et al., 2014). Interestingly, GROα and GROβ have been implicated in stimulating breast cancer cell metastasis and survival in the presence of chemotherapeutic agents (Acharyya et al., 2012). A combination of chemotherapy and CXCR2 inhibition effectively reduced the development of lung metastases after xenograft injections of human metastatic breast cancer cells into mice compared with either treatment alone (Acharyya et al., 2012).

Altogether, these data demonstrate the potential in therapeutically targeting CXCR2 in melanoma. Many CXCR2 inhibitors have been tested in animal and human trials for inflammatory conditions and have demonstrated positive effects and negligible toxicity (Stadtmann and Zarbock, 2012). CXCR2 antagonism has also demonstrated significant antitumor activity in a preclinical model for colon cancer (Ning et al., 2012) and slowed growth and antagonized metastasis in a recombinant mouse model of pancreatic adenocarcinoma (Shi et al., 2014). Alone, we found that a 30-mg/kg dose of navarixin in clear excess of that previously shown to inhibit neutrophil recruitment to sites of inflammation (<5 mg/kg; Chapman et al., 2007) was unable to halt the growth of implanted 4434 cells unless combined with MEKi. Also, we failed to detect a significant effect of navarixin alone at the doses tested on the growth of established human melanoma cells in culture, although CXCR2 knockdown by RNA interference did modestly diminish proliferation of A375 cells. Arguably, therefore, the concentration of navarixin in both in vivo and in vitro contexts was too low to completely ablate signaling. A dose of 100 mg/kg has demonstrated a significant effect on tumor growth in vivo as a single agent (Singh et al., 2009b), supporting this theory. What is clear from our data is the superadditive effect of combining MAPK and CXCR2 inhibitors in contexts where IL-1 signaling is active.

Blockade of IL-1 signaling was even more profound than CXCR2 antagonism at augmenting a growth inhibitory effect of MAPK inhibition in vivo and by itself had a marked effect on tumor growth. This might reflect the potency of gene ablation compared with drug antagonism or that IL-1 has pleiotropic effects on tumor growth in addition to initiating CXCR2 signaling in melanoma cells. Consistent with IL-1R1 expression on endothelial cells, IL-1 promotes tumor angiogenesis (Voronov et al., 2003), which is critical for tumor growth. IL-1 has also been implicated in immunosuppression in the tumor microenvironment through PDL1 induction in fibroblasts (Khalili et al., 2012). Therefore, IL-1 signaling could also be a promising candidate to target therapeutically. IL-1 blockade is used to treat a multitude of inflammatory diseases (Dinarello et al., 2012) and is generally well tolerated in patients (Mertens and Singh, 2009; Galloway et al., 2011). Clinical experience with IL-1R1 and CXCR2 antagonists should expedite translation of our findings.

We conclude that host cell activity in the melanoma microenvironment must be considered to develop the most effective therapeutic strategy for treating melanoma. Our study illustrates that a complex web of paracrine signals relayed between heterotypic cells within the tumor promotes treatment tolerance. We propose that targeting this network in parallel with MAPK inhibition would not only be extremely effective in reducing tumor growth, but also delay relapse in melanoma patients.

## Materials and methods

### Cell culture

All human melanoma cell lines and the 4434 *Braf^V600E^* mouse melanoma cell line (Table S1) as well as immortalized HFF cells (a gift from P. Caswell, The University of Manchester, Manchester, England, UK) were maintained in DMEM with l-glutamine, pyruvate, and sodium bicarbonate (Sigma-Aldrich) supplemented with 10% fetal bovine serum (Thermo Fisher Scientific) and 1% penicillin/streptomycin solution (Sigma-Aldrich). PBMCs from healthy donors were isolated from leukocyte cones (National Institute for Health Research Blood and Transplant) by subjecting to density gradient centrifugation using Ficoll Paque Plus (GE Healthcare) for 50 min at 400 RCF. PBMCs were transferred to flasks in serum-free RPMI 1640 Glutamax medium (Thermo Fisher Scientific) for 1 h at 37°C to allow enrichment of monocytes by adherence to tissue culture plastic. After differentiation to macrophages (see the Monocyte differentiation into macrophages section), cells were maintained in RPMI 1640 Glutamax medium (Thermo Fisher Scientific) supplemented with 10% fetal bovine serum and 1% penicillin/streptomycin solution. NHM cells were maintained in medium 254 (Thermo Fisher Scientific) supplemented with 1% human melanocyte growth supplement (Thermo Fisher Scientific). A375 CXCR2KD cells were generated by transfection (Lipofectamine; Invitrogen) using a previously described shRNA vector (Acosta et al., 2008), and clones were subsequently selected using puromycin. All cells were maintained under standard conditions at 37°C with 5% CO_2_.

### Patient samples

Patients with *BRAF^V600E^*-positive metastatic melanoma were treated with either a BRAFi or a combination of BRAFi and MEKi (details outlined in Table S2). All patients gave their consent for tissue acquisition according to an MD Anderson’s Institutional Review Board–approved protocol. Tumor biopsies were obtained before treatment (day 0), at 10–14 d on treatment, and/or at the time of progression, if applicable. Two commercially available cDNA arrays, MERT101 and MERT102 (OriGene), were analyzed for the expression of various genes in stage-III and stage-IV melanomas. The arrays consisted of cDNA derived from stage-III and stage-IV (*n* = 39) melanomas, staged according to the revised tumor nodes metastasis classification with minimum stage grouping (Balch et al., 2009), and from normal skin (*n* = 8). The expression in normal skin was set to one. β-Actin expression in each sample was used to normalize relative gene expression. Both these cDNA samples and the patient pretreatment and on-treatment cDNA samples had to be preamplified before qPCR analysis because of the low amount of cDNA provided. The cDNA samples were preamplified using the TaqMan PreAmp Master Mix kit (PN4384267; Applied Biosystems) using the following reaction mix: 25 µl of preamp master mix, 12.5 µl cDNA, and 12.5 µl of pooled primers (2.5 µl of each primer at 3 µM) in a 50-µl total reaction volume. All genes were amplified in the same reaction to ensure consistent preamplification. Samples were amplified using a G-Storm thermal cycler (GRI Ltd) and the following cycling conditions: 95°C for 15 s and 10 cycles at 60°C for 4 min. After preamplification, the reaction mix was diluted fivefold to generate a useable stock for qPCR.

### RNA isolation and qPCR analysis

RNA was isolated from samples using TRIzol (QIAGEN). cDNA was synthesized from RNA using the Omniscript reverse transcription kit (QIAGEN) according to the manufacturer’s instructions. Amplification of specific PCR products was detected using the SensiMix SYBR No-ROX kit (Bioline), an Mx3000P system (Agilent Technologies), and the following cycling conditions: 95°C for 10 min and 40 cycles at 95°C for 30 s, 55°C for 45 s, and 72°C for 45 s.

The following primer sequences were used for qPCR analysis: for human genes *β-actin* forward, 5′-GCAAGCAGGAGTATGACGAG-3′ and reverse, 5′-CAAATAAAGCCATGCCAATC-3′; *IL1A* forward, 5′-AATGACGCCCTCAATCAAAG-3′ and reverse, 5′-TGGGTATCTCAGGCATCTCC-3′; *TNFA* forward, 5′-TCAGAGGGCCTGTACCTCAT-3′ and reverse, 5′-GGAGGTTGACCTTGGTCTGG-3′; *IL10* forward, 5′-AAGACCCAGACATCAAGGCG-3′ and reverse, 5′-CACGGCCTTGCTCTTGTTTT-3′; *CD68* forward, 5′-TCAGCTTTGGATTCATGCAG-3′ and reverse, 5′-AGGTGGACAGCTGGTGAAAG-3′; *SMA* forward, 5′-ACCCACAATGTCCCCATCTA-3′ and reverse, 5′-GAAGGAATAGCCACGCTCAG-3′; *IL8* forward, 5′-GCTCAGTTTTGCCAAGGAGT-3′ and reverse, 5′-CTCTGCACCCAGTTTTCCTT-3′; and *CXCR2* forward, 5′-GCTCTTCTTCAGGGCACACT-3′ and reverse, 5′-ACCAGTGGACATGAGGC-3′; *IL1B* (Quantitect QT00021385; QIAGEN) and *GROα* (Quantitect QT00199752; QIAGEN). For mouse genes, *Gapdh* forward, 5′-TCTCCCTCACAATTTCCATCCCAG-3′ and reverse, 5′-GGGTGCAGCGAACTTTATTGATGG-3′; *Groα* (Quantitect QT00199752; QIAGEN); *Il1b* forward, 5′-ATGGCAACTGTTCCTGAACTCAACT-3′ and reverse, 5′-CAGGACAGGTATAGATTCTTTCCTTT-3′.

### Gene expression analysis using the Oncomine platform

The Oncomine dataset used in this study was the Talantov melanoma dataset (Talantov et al., 2005) containing 70 samples: 7 skin, 18 benign melanocytic skin nevi, and 45 cutaneous melanoma samples. The threshold settings were set as: P-value = 1E^−4^, fold-change = 2, and gene rank = top 10%. The dataset was exported from Oncomine and analyzed in Prism (GraphPad Software). 

### Monocyte differentiation into macrophages

After thorough washing, monocytes were incubated for 7 d in RPMI 1640 Glutamax medium with 10% fetal bovine serum and 1% penicillin/streptomycin solution supplemented with 100 ng/ml human M-CSF or Mel-CM to stimulate macrophage differentiation. To produce Mel-CM, melanoma cells were incubated in RPMI 1640 Glutamax medium for 72 h. Dead cells in the media were pelleted by centrifugation for 5 min at 200 RCF, and the media was subsequently filtered through a 0.45-µm filter. Conditioned media was diluted fourfold in fresh media before adding to culture flasks containing monocytes. On day 3 of incubation, 10 ml of fresh media (media supplemented with M-CSF or Mel-CM) was added to the culture flasks. Macrophages were detached by incubating with Accutase solution (Sigma-Aldrich) for 15 min followed by scraping and were subsequently seeded in tissue culture–coated plates. Cells were allowed to recover overnight before beginning assays. For the IFN-γ and LPS stimulation of macrophages, used as a positive control for IL-1β production in Fig. 1 (E and F), differentiated macrophages were stimulated with 100 ng/ml of human recombinant IFN-γ (PeproTech) for 24 h and then 20 ng/ml bacterial LPS (Sigma-Aldrich) for a further 24 h, which was directly added to the IFN-γ–supplemented media.

### ELISA

The level of IL-1β secretion by Mel-CM–treated macrophages 24 and 48 h after differentiation (when the cells were no longer in Mel-CM) and also by mouse Mel-CM–treated macrophages 24 h after stimulation was quantified with a Duoset ELISA (R&D Systems) according to the manufacturer’s instructions.

### IL-1β signaling blockade in fibroblasts and melanoma cell functional assay

HFF cells were cultured in conditioned media taken from NHM macrophages (NHM-Mϕ), A375-Mϕ, WM266-4-Mϕ, WM164-Mϕ, and MM485-Mϕ 24 h after the 7-d differentiation period (when the cells were no longer in conditioned media). The media was supplemented with 1 µg/ml normal goat IgG control (R&D Systems), 1 µg/ml IL-1β neutralizing antibody (R&D systems), or 1 µg/ml IL-1RA (PeproTech) overnight. The next morning, the cells were incubated in fresh media for 5 h, which was subsequently added to melanoma cells plated in 12-well plates with 1% DMSO (Sigma-Aldrich) or 0.5 µM both PLX4032 (Selleck Chemicals) and selumetinib (Selleck Chemicals). Then, HFF cell lysates were taken to analyze the expression of IL-6, IL-8, and GROα. After 48 h, melanoma cell survival was assayed by crystal violet staining (outlined in the Drug dose–response analysis and survival assays section).

### Melanoma cell survival assay with Fib-CM

Fully confluent fibroblasts plated in T162 flasks were treated overnight with either fresh media or fresh media supplemented with 100 ng/ml human recombinant IL-1β (PeproTech). The next morning, the cells were incubated in fresh media for 5 h, which was subsequently added to melanoma cells plated in either 6- or 12-well plates for 48 h with various inhibitors. The reagents used for these experiments were 1% DMSO (Sigma-Aldrich), 1 µM PLX4032 (Selleck Chemicals), 1 µM RAF265 (Selleck Chemicals), or 0.5 µM both PLX4032 and selumetinib (Selleck Chemicals). When the duotherapy treatment (PLX4032 and selumetinib) was also used in combination with either MK-2206, SB 225002, Bay 11-7082, or obatoclax, the concentration of each drug used was: 1 µM MK-2206 (Selleck Chemicals), 0.5 µM SB 225002 (Alfa Aesar), 0.2 µM Bay 11-7082 (Sigma-Aldrich), and 0.2 µM obatoclax (Selleck Chemicals). These concentrations were also used when these inhibitors were used as single agents. For each drug treatment, melanoma cells were cultured in nonconditioned media, conditioned media taken from unstimulated fibroblasts, or conditioned media taken from fibroblasts previously stimulated with IL-1β. Then, cell survival was assayed by crystal violet staining (outlined in the next section).

### Drug dose–response analysis and survival assays

For drug dose–responses assays, cells were plated in 96-well plates and treated with serial dilutions of PLX4032 (Selleck Chemicals) and selumetinib (Selleck Chemicals) for 72 h. For the melanoma cell survival assay with Fib-CM, melanoma cells were plated in either 6- or 12-well plates for 48 h with the various inhibitors as indicated in the figure legends. Cell survival was assayed by fixing and staining cells with 0.5% crystal violet in 4% formaldehyde. Survival was quantified by measuring the absorbance of the solubilized dye (in 2% SDS in PBS) at an optical density of 595 nm.

### Cytokine array

IMR-90 human diploid fibroblasts were transduced with empty MSCV-puro retroviral vector or vector encoding IL-1A as previously described (Acosta et al., 2013). IMR-90 cells were selected with puromycin 48 h after infection at a final concentration of 0.5 mg/ml for 1 wk. For the antibody array, supernatant was harvested from cells and passed through a 0.2-µm filter to remove cells before being incubated with cytokine V arrays (RayBiotech) according to the manufacturer’s instructions. Signal on the membrane was developed using enhanced chemiluminescence and scanned. Scanned images were quantified using ImageJ software (National Institutes of Health).

### Macrophage generation and function in *Il-1r1^−/−^* mice

#### Isolation and stimulation

*Il-1r1^−/−^* flox control and knockout mice have been previously described (Abdulaal et al., 2016) and were provided by A. Waisman (University of Mainz, Mainz, Germany), W. Muller, and E. Pinteaux (The University of Manchester, Manchester, England, UK). Bone marrow cell suspensions were collected from femurs and tibias of 8–15-wk-old mice by flushing with complete DMEM (10% FBS and 1% penicillin/streptomycin solution) using Myjector U-100 insulin syringes with 29G × 0.5 needles. Cell aggregates were resuspended by gentle pipetting, and the solution was passed through a 40-µm nylon web. After centrifugation, cells were resuspended in complete DMEM supplemented with 15% l-929 cell–conditioned medium (as a source of M-CSF) to induce macrophage differentiation. Cells were seeded on 12- or 6-well ultra-low attachment surface plates (Corning) and cultured in a humidified incubator at 37°C and 5% CO_2_. At day 7, differentiated macrophages were washed and incubated with complete DMEM (control) or 100 ng/ml LPS (Sigma-Aldrich) and 50 ng/ml IFN-γ (PeproTech) or with 4434 melanoma– or NIH3T3 fibroblast–conditioned supernatant. After 24 h, cells were washed and incubated with complete DMEM for 24 h. Macrophage-conditioned media was collected and analyzed by ELISA to detect mature secreted IL-1β as described in the ELISA section.

#### Flow cytometry

Single-cell suspensions of bone marrow mononuclear cells were analyzed on a FACS. Cell suspensions were pelleted, washed twice, and resuspended in magnetic-activated cell-sorting solution (PBS containing 10% FBS and 1 mM EDTA). A trypan blue exclusion viability test was performed to discriminate dead from live cells. For surface staining, cells were first incubated with anti–mouse FcR antibody (mouse seroblock FcR; BioRad Laboratories) for 20 min at 4°C. Then, mononuclear cells were stained with the following antibodies from BD, conjugated to either FITC or PE: CD115-PE (1:80) and F4/80-FITC (1:100). Flow cytometry analysis was performed with a FACScan instrument (BD) and analyzed using FlowJo software (Tree Star).

### Mouse allograft model

4434 subcutaneous implantation was performed as previously described (Smith et al., 2014). Treatment commenced when tumors reached 100 mm^3^, and mice were randomly assigned into groups. Both drugs were prepared in 8:1:1 (vol/vol/vol) water/ethanol/Cremophor EL (Sebolt-Leopold et al., 1999). The CXCR2 inhibitor navarixin (SCH 527123; MK-7123) was dosed at 30 mg/kg at 0.1 ml/10 g body weight, and the MEKi PD184352 was dosed at 25 mg/kg, by oral gavage once daily. Mice were weighed and measured every other day until the tumor reached 800 mm^3^. Then, tumors were harvested and snap frozen for mRNA analysis or were formalin fixed and paraffin embedded for immunohistochemistry. All animal procedures involving animals were ethically approved by The University of Manchester Animal Welfare and Ethical Review Body and performed under license in accordance with the UK Home Office Animals (Scientific Procedures) Act of 1986 and guidelines of the Committee of the National Cancer Research Institute (Workman et al., 2010).

### Immunoblotting and immunohistochemistry

Cells were lysed in SDS lysis buffer and analyzed using standard Western blotting protocols. Scanned Western blot images were quantified using ImageJ software. Formalin-fixed paraffin-embedded tissue blocks used for this study were retrieved from the archive of the Department of Pathology, Spedali Civili di Brescia. Human tissues included primary cutaneous melanoma and skin and lung metastasis of primary cutaneous melanoma. 4 µm–thick tissue sections were used for immunohistochemical staining. For double and triple staining, after completing the first immune reaction, the second reaction was visualized using Mach 4 MR-AP (Biocare Medical), followed by Ferangi Blue. For triple staining, the third reaction was revealed using a REAL Detection System (Alkaline Phosphatase/RED Rabbit/Mouse; Dako).

### Antibodies

The antibodies used for immunoblot analysis included IL-1β (1:1,000; R&D Systems), GROα (1:1,000; Thermo Fisher Scientific), IL-6 (1:1,000; R&D Systems), IL-8 (1:1,000; R&D Systems), IL-1R1 (1:1,000; R&D Systems), phosphorylated ERK (pERK; 1:5,000; Sigma-Aldrich), β-tubulin (1:5,000; Santa Cruz Biotechnology, Inc.), ERK2 (1:5,000; Santa Cruz Biotechnology, Inc.), pp65 (1:1,000; Cell Signaling Technology), p65 (1:1,000; Cell Signaling Technology), and BCL2 (1:1,000; Cell Signaling Technology). Anti–rabbit IgG-HRP (1:5,000) and anti–mouse IgG-HRP (1:5,000) were obtained from GE Healthcare, and anti–goat IgG-HRP (1:2,000) was obtained from Santa Cruz Biotechnology, Inc. The primary antibodies used for immunohistochemical analysis included anti–IL-1β (goat polyclonal; 1:50; R&D Systems), anti–IL-1R1 (goat polyclonal; 1:50; R&D Systems), anti-CD68 (clone KP1; mouse; 1:300; Dako), anti-CD163 (clone 10D6; mouse; 1:50; Thermo Fisher Scientific), anti-SOX10 (goat polyclonal; 1:120; Santa Cruz Biotechnology, Inc.), anti–IBA-1(rabbit polyclonal; 1:300; Wako Pure Chemical Industries), anti-SMA (clone 1A4; mouse; 1:200; Thermo Fisher Scientific), and anti-GROα (rabbit polyclonal; 1:50; Proteintech) followed by appropriate detection systems.

### Statistical analysis

Data were analyzed using a one-way ANOVA followed by a posthoc test (Dunn’s or Tukey’s multiple comparisons, as indicated in the figure legends), a Mann-Whitney test, or a Student’s *t* test, as indicated, using Prism (version 6; GraphPad Software). Pearson correlation was used to analyze associated gene expression.

### Online supplemental material

Table S1 shows the origin and mutational status of human melanoma cell lines used in this study. Table S2 shows patient characteristics.

## Supplementary Material

Supplemental Materials (PDF)
