# Supplementary material for: An adaptive signaling network in melanoma inflammatory niches confers tolerance to MAPK signaling inhibition
Source: J Exp Med. 2017 Jun 5;214(6):1691–710. doi: 10.1084/jem.20160855 (PMC5460994; doi:10.1084/jem.20160855)
Supplement: Supplemental Materials (PDF) [file JEM_20160855_sm.pdf]

The Journal of Experimental Medicine

SUPPLEMENTAL MATERIAL

Young et al., https://doi.org/10.1084/jem.20160855

Table S1. **The origin and mutational status of human melanoma cell lines used in this study**

Cell line	Stage	BRAF	PTEN	NRAS
WM266-4	MET	V600D	Homozygous deletion	WT
WM164	MET	V600E	WT	WT
A375	MET	V600E	WT	WT
888mel	MET	V600E	WT	WT
WM1361	VGP	WT	WT	Q61K
WM852	MET	WT	Hemizygous deletion	Q61R
MM485	MET	WT	WT	Q61R
DO4	VGP	WT	WT	Q61L
MV3	RGP	WT	Unknown	WT
501mel	MET	V600E	WT	WT
LU1205	Xenograft MET	V600E	Hemizygous deletion/Mut W274X	WT

Stage of melanoma progression at which biopsies were taken: radial growth phase (RGP), vertical growth phase (VGP), or from either a lymph node or secondary site metastasis (MET). Xenograft metastases were cells passaged in mice to isolate highly metastatic cells. BRAF, phosphatase and tensin homolog (PTEN), and neuroblastoma RAS viral oncogene homolog (NRAS) mutational status is shown.

Table S2. **Patient characteristics**

Patient	Mutation	Treatment	Response/max	Time to progression
				mo
3	BRAF	BRAFi	SD (−10%)	10
4	BRAF	BRAFi	PR (−56%)	3.5
8	BRAF	BRAFi + MEKi	PR (−30%)	3
9	BRAF	BRAFi + MEKi	PR (−45%)	7
11	BRAF	BRAFi + MEKi	PR (−80%)	10
12	BRAF	BRAFi + MEKi	PR (−88.9%)	12, stopped at 20
13	BRAF	BRAFi + MEKi	PR (−57.9%)	9, stroke
18	BRAF	BRAFi + MEKi	SD (−16.5%)	6
24	BRAF	BRAFi	PR (−53%)	2
25	BRAF	BRAFi + MEKi	PR (−64%)	3

Patients with metastatic melanoma all harboring a $BRAF^{V600E}$ mutation (confirmed by genotyping) were enrolled on clinical trials for treatment with vemurafenib (BRAFi) or a combination of dabrafenib and trametinib (BRAFi/MEKi). Patient, treatment, maximal response, and duration of response are reported. PR, partial response; SD, stable disease.
